# Temporal, Spatial, and Genomic Analyses of *Enterobacteriaceae* Clinical Antimicrobial Resistance in Companion Animals Reveals Phenotypes and Genotypes of One Health Concern

**DOI:** 10.3389/fmicb.2021.700698

**Published:** 2021-07-30

**Authors:** David A. Singleton, Pisut Pongchaikul, Shirley Smith, Rebecca J. Bengtsson, Kate Baker, Dorina Timofte, Stephen Steen, Matthew Jones, Larry Roberts, Fernando Sánchez-Vizcaíno, Susan Dawson, P.-J. M. Noble, Alan D. Radford, Gina L. Pinchbeck, Nicola J. Williams

**Affiliations:** ^1^Institute of Infection, Veterinary and Ecological Sciences, University of Liverpool, Neston, United Kingdom; ^2^Chakri Naruebodindra Medical Institute, Faculty of Medicine Ramathibodi Hospital, Mahidol University, Salaya, Thailand; ^3^NationWide Laboratories/C.A.P.L. Ltd., Knutton, United Kingdom; ^4^IDEXX Laboratories Ltd., Wetherby, United Kingdom; ^5^Bristol Veterinary School, University of Bristol, Bristol, United Kingdom

**Keywords:** antimicrobial resistance, companion animal, surveillance, digital health, *Escherichia coli*, one health

## Abstract

**Background:**

Antimicrobial resistance (AMR) is a globally important one health threat. The impact of resistant infections on companion animals, and the potential public health implications of such infections, has not been widely explored, largely due to an absence of structured population-level data.

**Objectives:**

We aimed to efficiently capture and repurpose antimicrobial susceptibility test (AST) results data from several veterinary diagnostic laboratories (VDLs) across the United Kingdom to facilitate national companion animal clinical AMR surveillance. We also sought to harness and genotypically characterize isolates of potential AMR importance from these laboratories.

**Methods:**

We summarized AST results for 29,330 canine and 8,279 feline *Enterobacteriaceae* isolates originating from companion animal clinical practice, performed between April 2016 and July 2018 from four VDLs, with submissions from 2,237 United Kingdom veterinary practice sites.

**Results:**

*Escherichia coli* (*E. coli*) was the most commonly isolated *Enterobacteriaceae* in dogs (69.4% of AST results, 95% confidence interval, CI, 68.7–70.0) and cats (90.5%, CI 89.8–91.3). Multi-drug resistance was reported in 14.1% (CI 13.5–14.8) of canine and 12.0% (CI 11.1–12.9) of feline *E. coli* isolates. Referral practices were associated with increased *E. coli* 3rd generation ≤ cephalosporin resistance odds (dogs: odds ratio 2.0, CI 1.2–3.4). We selected 95 *E. coli* isolates for whole genome analyses, of which seven belonged to sequence type 131, also carrying the plasmid-associated extended spectrum β-lactamase gene *bla*_CTX–M–__15_. The plasmid-mediated colistin resistance gene *mcr-9* was also identified for the first time in companion animals.

**Conclusions:**

Linking clinical AMR data with genotypic characterization represents an efficient means of identifying important resistance trends in companion animals on a national scale.

## Introduction

Antimicrobial resistance (AMR) poses a significant global threat to animal and human health (O’Neill, 2016). The World Health Organization (WHO) has identified *Enterobacteriaceae* to be of critical importance, due to worldwide dissemination of extended spectrum β-lactamases (ESBLs), ampicillin hydrolyzing (AmpC) β-lactamases, and carbapenemases (Iredell et al., 2016; Bezabih et al., 2021; Salgado-Caxito et al., 2021). Additionally, newly emerging resistance trends, for example mobile colistin resistance (Elbediwi et al., 2019; El-Sayed Ahmed et al., 2020), have led to increased reports of multi-drug resistant (Magiorakos et al., 2012) and pan-resistant strains (Sun et al., 2016).

To address these issues, national AMR surveillance in humans and production animals has been established (VMD, 2019; PHE, 2020). However, companion animal species have been arguably neglected, despite resistance developing in companion animals exposed to antimicrobial therapy (Schmidt et al., 2018), and transmission of pathogens and resistance between humans and companion animals (Lei et al., 2017). This oversight has partly been due to the companion animal veterinary diagnostic laboratory (VDL) sector being largely comprised of independent commercial laboratories, each utilizing often bespoke information management systems (O’Neill et al., 2014), and different protocols for antimicrobial susceptibility testing (AST) (Marques et al., 2016). Such protocols largely focus on relatively time-consuming techniques for demonstrating phenotypic resistance. Recently, genomics-based methodologies have unlocked a wealth of information pertaining to the development and dissemination of resistance genes in and between bacterial species (McCarthy et al., 2015). Though this has only begun to be explored in companion animals (Zhang, 2016), bacterial isolates disposed of by VDLs remain an untapped resource.

Hence, the aim of this study was, using *Enterobacteriaceae* isolated from clinical samples of dogs and cats as an exemplar, to define national patterns of AST in companion animals, and factors associated with resistance. Further, we sought to develop a method by which *Enterobacteriaceae* isolates expressing resistance patterns of potential public health importance could be efficiently captured from VDLs for genotypic characterization. We also sought to investigate the AST method and interpretation variation between VDLs, and to outline how such variation might impact on more detailed genotypic investigations as performed here.

## Materials and Methods

### Antimicrobial Susceptibility Test Data

Clinical AST data were collated by the Small Animal Veterinary Surveillance Network (SAVSNET) from four United Kingdom VDLs (A–D) between 1st April 2016 and 31st July 2018, including a unique sample identifier; submitting veterinary practice site postcode; report date; animal species; breed; sex and neuter status; bacterial species isolated, and AST results. Sample type/site (supplied as free text) was manually categorized into urine, ear(s), oro-nasopharyngeal and respiratory sites, feces, anal region including anal sacs, and other/mixed sites. SAVSNET holds ethical approval to collect such data from the University of Liverpool Ethics Committee (RETH0000964).

The manner with which bacterial species were described varied between VDLs, ranging from genus-level (e.g., *Escherichia*) alone to species-level (e.g., *Escherichia coli*). Similarly, bacteria were speciated according to varying methodologies, ranging from commercial biochemical tests (VDLs B–D) to MALDI-TOF (VDL A); *Enterobacteriaceae* were selected for further analysis. Laboratories utilized disk diffusion (VDL B-D) or minimum inhibitory concentration (MIC) (VDL A) approaches, and used different interpretation guidelines (CLSI, EUCAST, and BSAC). As some VDLs (B–D) only provided clinically interpreted AST results, analysis was restricted to interpreted results alone. Only antimicrobials associated with species-specific acquired resistance were summarized. Tested antimicrobials were grouped to class-level (Supplementary Material 1); ‘intermediate’ results were classified as ‘sensitive,’ with multi-drug resistance (MDR) defined as resistance to three or more classes (Magiorakos et al., 2012).

### Veterinary Practice Site Demographic Data

The Royal College of Veterinary Surgeons (RCVS) Veterinary Practice Directory (RCVS, 2018) was interrogated on 12th October 2018, using R packages ‘rvest’ (Wickham, 2016) and ‘purrr’ (Henry, 2018), to extract information for all United Kingdom veterinary practice sites (Table 1); postcodes containing more than one site treating companion animals (*n* = 148) were designated ‘multi-site postcodes.’ The National Statistics Postcode Look-up was used to provide geographical information, including urban/rural status (NSPL, 2015).

**TABLE 1:** Summary of the percentage of *Enterobacteriaceae* antimicrobial susceptibility test results originating from a range of veterinary practice site categories, as defined by the Royal College of Veterinary Surgeons (RCVS) on 12th October 2018, compared against the percentage of veterinary practice sites in the full RCVS practice register.

**Variable**	**Category**	**Dog**	**Cat**	**RCVS practice register**
	
		**%^a^ (95% CI^b^)**	**% (95% CI)**	**% of practice sites (95% CI)**
Species treated	Dogs and cats	74.9 (72.2–77.6)	81.0 (78.1–83.9)	80.0 (78.8–81.2)
	Dogs, cats, and equids	2.5 (1.5–3.5)	2.3 (1.3–3.4)	2.8 (2.3–3.2)
	Dogs, cats, and farmed species	3.0 (1.9–4.1)	2.6 (1.4–3.8)	2.1 (1.7–2.6)
	Mixed	12.7 (10.7–14.8)	8.3 (6.3–10.2)	15.1 (14.1–16.1)
RCVS^c^ accreditation	Accredited sites	49.8 (46.7–53.0)	52.9 (48.8–57.1)	42.5 (41.1–44.0)
RCVS veterinary hospital	Sites with hospital status	17.5 (14.4–20.7)	19.3 (14.5–24.1)	4.6 (4.0–5.2)
Out of hours (OOH) providers	Sites providing OOH only	5.5 (3.6–7.5)	4.9 (3.1–6.8)	2.3 (1.8–2.7)
Referrals	Sites providing referrals only	1.8 (0.6–2.9)	1.7 (0.6–2.9)	1.3 (1.0–1.6)
	First opinion and referral sites	1.2 (0.4–1.9)	1.1 (0.2–1.9)	0.8 (0.5–1.0)
RCVS AVP^d^	Sites employing 1 < AVP	23.3 (20.2–26.4)	25.7 (21.0–30.4)	13.6 (12.6–14.6)
RCVS specialist	Sites employing 1 < specialist	7.1 (4.9–9.4)	8.7 (4.3–13.0)	2.6 (2.1–3.1)
Veterinary nurse (VN) training sites	Sites training VNs	79.0 (76.8–81.2)	80.0 (77.2–82.8)	56.6 (55.1–58.1)
Location	Urban	77.5 (74.9–80.1)	84.2 (81.4–86.9)	71.9 (70.6–73.2)
RCVS practice register match	No match^e^	6.7% (5.3–8.1)	5.8% (4.1–7.4)	–
	Match to >1 site^f^	3.9% (2.5–5.3)	3.6% (2.2–5.0)	–

*^a^Percentage of *Enterobacteriaceae* AST results.*

*^b^95% Confidence interval.*

*^c^Royal College of Veterinary Surgeons.*

*^d^Advanced veterinary practitioner.*

*^e^Results associated with a postcode not matching the RCVS practice registry.*

*^f^Results associated with a postcode registered to two or more companion animal-treating sites.*

### Animal Demographic Data

Dog and cat breeds were summarized to standardized breed terms, which were then grouped into genotypically similar groups. Three further breed groups were defined: crossbreeds, breeds not yet genetically classified (‘unclassified’), and breeds not recorded or recognizable (‘unknown’) (Singleton et al., 2020).

### Multidrug Resistant Isolate Collection and Storage

Six VDLs (A–F) were approached regarding collection of *Enterobacteriaceae* isolates, of which three (A, E, F) participated. Submitted isolates needed to comply with one or more of the following criteria: (1) Phenotypic ESBL-producer, (2) MDR, or (3) non-susceptibility to one or more of the 3rd/4th generation cephalosporin, fluoroquinolone, polymyxin, or carbapenem classes. On receipt at the University of Liverpool, isolates (*n* = 148) were incubated under aerobic conditions for 18–24 h at 37°C on nutrient agar, and pure colonies stored at −80°C (Microbank^TM^ cryovial; Pro-Lab Diagnostics, United Kingdom).

### Phenotypic Analyses

To mitigate for protocol variation between VDLs, disk diffusion ASTs were undertaken on all received isolates according to CLSI guidelines (CLSI, 2008). Antimicrobial disks (MAST Diagnostics; Liverpool, United Kingdom) represented a range of antimicrobial classes (Supplementary Material 2). Inhibition zones were measured after aerobic incubation at 37°C overnight, and classified as susceptible, intermediate or resistant, or ESBL-producing via a combination disk method (Supplementary Material 2; MAST Diagnostics; Liverpool, United Kingdom) (CLSI, 2008) ‘Intermediate’ results were considered ‘sensitive.’

### Genotypic Analyses

Crude nucleic acid extracts were prepared prior to PCR amplification, with isolates classified as *E. coli* via presence of the *uidA* (McDaniels et al., 1996) or *uspA* genes (Bekal et al., 2003) (Supplementary Material 3). All isolates were tested for *bla*_CTX–M_ (Boyd et al., 2004), with positive isolates characterized into *bla*_CTX–M_ cluster groups 1, 2, or 9 (Batchelor et al., 2005; Hopkins et al., 2006; Carattoli et al., 2008). Isolates possessing *bla*_CTX–M_ group 1 genes were characterized into *bla*_CTX–M–__1_ or *bla*_CTX–M–__15_ variants via real-time PCR (Isgren et al., 2019). All isolates were tested for presence of *bla*_TEM_, *bla*_SHV_ or *bla*_OXA_ (Dallenne et al., 2010), and those displaying potential AmpC phenotypes for *bla*_CIT_, *bla*_DHA_, *bla*_ACC_, *bla*_EBC_ and *bla*_FOX_ (Perez-Perez and Hanson, 2002). All isolates were tested for presence of *qnrA*, *qnrB*, or *qnrS*, genes (Robicsek et al., 2006).

*E. coli* isolates were selected for whole genome sequencing (WGS) based on evidence of phenotypic ESBL production; MDR status; presence of plasmid-associated AmpC, or plasmid-associated fluoroquinolone resistance genes on PCR. Fragment libraries (NEBNext Ultra II FS Kit; ∼300 base pair inserts) were created from purified genomic DNA (Qiagen QIAmp DNA mini kit) and sequenced using a 2 × 150 base pair paired-end protocol (Illumina HiSeq; CGR, University of Liverpool; GenBank accession numbers: Supplementary Material 4). *De novo* assembly was performed using SPAdes 3.16.0 (Bankevich et al., 2012), and genome annotation via Prokka 1.14.5 (Seemann, 2014). *In silico* identification of MLST and AMR genes were analyzed via MLST 2.19.0 (Seemann, 2019), ABRicate 0.8 (Seemann, 2020a), and BLASTP searches. Core genome SNPs were determined via Snippy 4.4.5 (Seemann, 2020b); phylogenies were estimated via maximum likelihood phylogenetic analysis using raxml-ng (Kozlov et al., 2019), with a generalized time-reversible model and 1,000 bootstraps, and annotated using iTOL 4 (Letunic and Bork, 2019). Plasmids were identified via PlasmidFinder (Carattoli et al., 2014), summarizing incompatibility (Inc) group plasmids with 100% coverage and >80% identity.

### Statistical Analyses

All statistical analyses were completed using R (v3.4.4). Descriptive proportions and 95% confidence intervals were adjusted for clustering within practice sites (bootstrap method, *n* = 5,000 samples) (AOD, 2016). Mixed effects logistic regression models exploring odds of *E. coli* resistance were fitted separately in dogs and cats (LME4, 2016), considering 3rd/4th generation cephalosporin resistance as a binary outcome variable. MDR, fluoroquinolone and potentiated penicillin resistance were also modeled (Supplementary Materials 5–13). Both practice site and VDL were included as random effects if a likelihood ratio test (LRT) suggested improved fit. Initial univariable screening considered a range of categorical explanatory variables, including sample type/site and animal or practice site demographic features (Supplementary Material 8). Explanatory variables were retained for multivariable analysis if a LRT indicated *P* ≤ 0.20 against a null model.

Multivariable models underwent step-wise backward elimination to minimize Akaike Information Criterion (AIC). Two-way interaction terms were retained if inclusion resulted in an AIC decrease. Multicollinearity was assessed using the Variance Inflation Factor (CAR, 2018). Statistical significance was defined as *P* < 0.05.

## Results

### AST Data – Laboratories A–D

Data were obtained for 29,330 canine and 8,279 feline *Enterobacteriaceae* isolates from 2,237 practice sites, corresponding to 49.1% (95% confidence interval, CI, 47.7–50.6) of registered companion animal-treating sites (Supplementary Material 14), contributing a median of seven canine (range 1–156) and three feline (range 1–166) isolates each. Though practice site details could not be ascertained in 6.7% (CI 5.3–8.1) of canine and 5.8% (CI 4.1–7.4) of feline AST reports, site demographic and animal population features of the remainder are summarized in Table 1 and Supplementary Material 15, respectively.

In total, 65 *Enterobacteriaceae* species were isolated (Supplementary Material 16). *E. coli* was most common, representing 69.4% (CI 68.7–70.0) of canine, and 90.5% (CI 89.8–91.3) of feline AST results, with urine being the most recorded sample type/site (58.6% of canine and 83.8% of feline *E. coli* isolates; Supplementary Material 17). MDR *E. coli* were reported in 14.1% of canine (CI 13.5–14.8) and 12.0% (CI 11.1–12.9) of feline isolates, MDR *Proteus mirabilis* in 3.1% (CI 2.5–3.6) of canine, and 7.1% (CI 4.0–10.1) of feline isolates, and MDR *Klebsiella pneumoniae* in 21.3% (CI 15.3–27.4) of canine and 38.5% (CI 20.1–56.9) of feline isolates (Table 2).

**TABLE 2:** Summary of canine and feline *E. coli, P. mirabilis*, and *K. pneumoniae* clinical antimicrobial class-level resistance.

**Antimicrobial class**	***E. coli***			
	**Dogs**		**Cats**	
	**%^a^ (CI)^b^**	***n* tested**	**% (CI)**	***n* tested**
Resistant to 1 or more classes	34.8 (34.0–35.5)	20343	29.8 (28.7–31.0)	7497
Resistant to 1 or 2 classes	20.6 (20.0–21.2)	20343	17.8 (16.9–18.7)	7497
Resistant to 3 or more classes	14.1 (13.5–14.8)	20343	12.0 (11.1–12.9)	7497
Aminoglycosides	2.4 (2.0–2.8)	7583	1.9 (0.9–3.0)	1141
Amphenicols	5.3 (4.1–6.5)	1657	5.0 (0.9–9.1)	180
Nitrofurantoin	0.7 (0.6–0.9)	11103	0.4 (0.2–0.5)	5845
Polymyxins	2.4 (0.3–4.4)	336	0.0	24
Fluoroquinolones	5.5 (5.0–6.0)	20341	2.2 (1.6–2.7)	7497
Potentiated sulphonamides	10.1 (9.5–10.7)	18734	3.9 (3.3–4.5)	7339
Tetracyclines	11.8 (11.2–12.4)	18016	5.2 (4.6–5.8)	7189
Beta-lactams	33.3 (32.5–34.2)	20343	29.3 (28.1–30.5)	7497
*Extended spectrum penicillins*	34.5 (33.5–35.4)	18654	28.8 (27.6–30.0)	7327
*1st/2nd gen. cephalosporins*	13.3 (12.7–13.9)	20282	13.2 (12.3–14.1)	7484
*3rd/4th gen. cephalosporins*	8.4 (7.9–9.0)	17020	7.2 (6.5–8.0)	6730
*Potentiated penicillins*	12.7 (12.1–13.4)	20293	11.7 (10.8–12.6)	7489
*Carbapenems*	0.0	75	0.0	12

	***P. mirabilis***			

Resistant to 1 or more classes	26.0 (24.8–27.2)	5326	33.9 (28.3–39.6)	268
Resistant to 1 or 2 classes	22.9 (21.8–24.1)	5326	26.9 (21.5–32.4)	268
Resistant to 3 or more classes	3.1 (2.5–3.6)	5326	7.1 (4.0–10.1)	268
Aminoglycosides	3.3 (2.5–4.1)	2017	4.2 (0.0–8.8)	72
Amphenicols	8.5 (6.9–10.1)	1359	8.4 (0.0–17.4)	36
Fluoroquinolones	4.0 (3.4–4.7)	5326	7.1 (3.7–10.5)	266
Potentiated sulphonamides	13.6 (12.4–14.8)	3972	18.7 (13.6–23.8)	230
Beta-lactams	18.0 (16.9–19.1)	5326	26.1 (20.8–31.4)	268
*Extended spectrum penicillins*	21.8 (20.4–23.2)	3968	28.5 (22.8–34.3)	231
*1st/2nd gen. cephalosporins*	4.6 (4.0–5.2)	5323	8.2 (4.8–11.6)	268
*3rd/4th gen. cephalosporins*	1.6 (1.2–2.1)	3961	3.1 (0.9–5.2)	231
*Potentiated penicillins*	0.1 (0.0–0.2)	4963	0.0	243

	***K. pneumoniae***			

Resistant to 1 or more classes	31.3 (25.2–37.4)	483	46.6 (28.0–65.2)	48
Resistant to 1 or 2 classes	10.1 (7.4–12.9)	483	8.3 (9.8–15.6)	48
Resistant to 3 or more classes	21.3 (15.3–27.4)	483	38.5 (20.1–56.9)	48
Aminoglycosides	4.7 (2.2–7.2)	274	11.6 (0.0–26.6)	34
Amphenicols	9.3 (0.9–17.7)	53	0.0	9
Nitrofurantoin	33.3 (25.9–40.7)	201	6.1 (0.0–17.8)	16
Fluoroquinolones	18.5 (13.4–23.7)	483	32.2 (15.0–49.4)	48
Potentiated sulphonamides	13.0 (7.7–18.2)	446	33.6 (17.7–53.0)	43
Tetracyclines	17.4 (12.1–22.6)	426	35.3 (17.7–53.0)	41
Beta-lactams	24.4 (18.4–30.4)	483	42.3 (23.0–61.7)	48
*1st/2nd gen. cephalosporins*	24.0 (18.0–30.0)	483	40.1 (20.4–59.8)	48
*3rd/4th gen. cephalosporins*	20.0 (13.8–26.1)	412	41.0 (21.8–60.2)	40
*Potentiated penicillins*	21.2 (15.1–27.2)	483	38.5 (20.4–56.6)	48
*Carbapenems*	0.0	6	0.0	3

*^a^Percentage of tested isolates resistant.*

*^b^95% confidence interval.*

#### 3rd/4th Generation Cephalosporin Resistant *E. coli*

**FIGURE 1:**
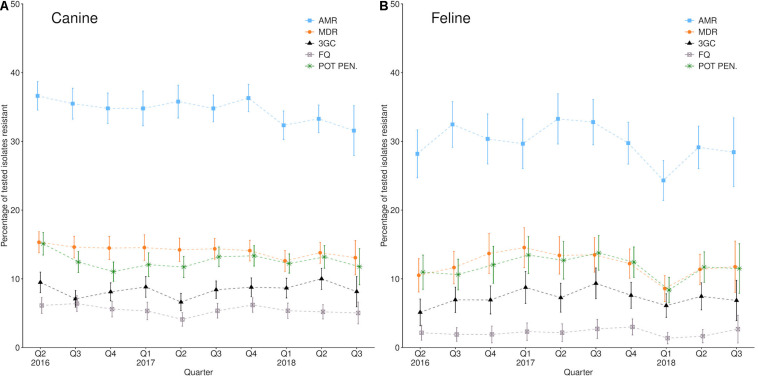
Percentage of **(A)** canine and **(B)** feline *Enterobacteriaceae* AST results reporting single-class resistance (AMR); multi-drug resistance (MDR); 3rd/4th generation cephalosporin resistance (3GC); fluoroquinolone resistance (FQ), or potentiated penicillin resistance (POT PEN.) between the 2nd quarter of 2016 and the third quarter of 2018. Error bars refer to 95% confidence intervals.

**FIGURE 2:**
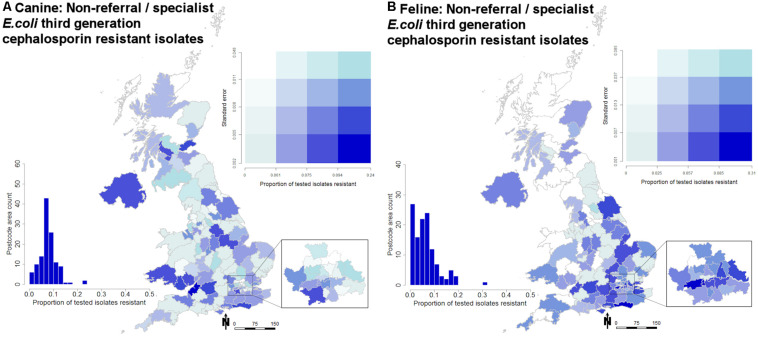
Quartile bivariate postcode map displaying the proportion of tested isolates that were submitted by non-referrals/specialist veterinary practice sites recording phenotypic 3rd/4th generation cephalosporin resistance in **(A)** dogs and **(B)** cats. Proportions are displayed against standard error. Scale is in Km. The embedded histogram displays the proportion of tested isolates reporting phenotypic 3rd/4th generation cephalosporin resistance against a count of postcode areas (*n* = 124).

While prevalence remained relatively stable over the 2 years summarized (Figure 1), geographical variation was observed (Figure 2). Male dogs were associated with reduced odds of 3rd/4th generation cephalosporin resistance, whereas male cats were associated with increased odds (Table 3). Practice sites examining referral cases only were associated with increased odds, as were RCVS accredited sites. Compared to urine samples, the anal region and oronasopharyngeal/respiratory samples were associated with increased odds in dogs and cats, respectively (univariable findings, Supplementary Materials 18,19).

**TABLE 3:** Multivariable mixed effects logistic regression models displaying risk factors significantly associated with odds of an *E. coli* clinical isolate being classed as 3rd/4th generation cephalosporin resistant in dogs and cats.

**Variable**	**Category**	**β**	***SE*^a^**	**OR^b^ (CI)^c^**	***P***
**Dogs**					
Intercept		−2.45	0.59	0.09 (0.04–0.27)	
Sex	Female	–	–	1.00	–
	**Male**	−**0.13**	**0.06**	**0.88 (0.78–0.99)**	**0.03**
	Unspecified	0.16	0.16	1.18 (0.87–1.60)	0.30
Genetic breed group^d^	Retriever	–	**–**	1.00	**–**
	**Ancient/spitz**	**0.46**	**0.22**	**1.59 (1.03–2.43)**	**0.04**
	**Crossbreed**	**0.28**	**0.11**	**1.32 (1.06–1.64)**	**0.01**
	Herding	−0.12	0.17	0.89 (0.64–1.23)	0.47
	**Mastiff-like**	**0.35**	**0.12**	**1.41 (1.11–1.79)**	**<0.01**
	**Scent hound**	**0.50**	**0.17**	**1.65 (1.18–2.29)**	**<0.01**
	Sight hound	−0.36	0.29	0.70 (0.40–1.22)	0.21
	**Small terrier**	**0.46**	**0.13**	**1.58 (1.21–2.05)**	**<0.01**
	**Spaniel**	**0.26**	**0.11**	**1.30 (1.04–1.62)**	**0.02**
	**Toy**	**0.46**	**0.17**	**1.59 (1.14–2.20)**	**0.01**
	Not yet genetically classified	0.14	0.13	1.15 (0.90–1.47)	0.27
	Unknown breed	0.22	0.12	1.25 (0.98–1.59)	0.07
	**Working dog**	**0.44**	**0.15**	**1.56 (1.17–2.08)**	**<0.01**
Species treated	Dog and cat	–	–	1.00	–
	Dog, cat, and equine	0.19	0.20	1.21 (0.82–1.76)	0.34
	Dog, cat, equine, and farm	−0.20	0.11	0.82 (0.66–1.01)	0.06
	Dog, cat, and farm	0.12	0.19	1.13 (0.78–1.65)	0.53
RCVS accreditation	Not accredited	–	–	1.00	–
	**Accredited**	**0.14**	**0.07**	**1.15 (1.01**–**1.32)**	**0.03**
Referrals only	Not referrals-only site	–	–	1.00	–
	**Referrals-only site**	**0.70**	**0.27**	**2.02 (1.20**–**3.42)**	**0.01**
	Mixed site	−0.34	0.33	0.71 (0.37–1.36)	0.31
RCVS Specialist	No RCVS specialist on site	–	–	1.00	–
	**RCVS specialist on site**	**0.43**	**0.15**	**1.54 (1.16**–**2.06)**	**<0.01**
Sampling type/site	Urine	–	–	1.00	–
	**Anal region (including anal sacs)**	**0.24**	**0.09**	**1.28 (1.08**–**1.51)**	**0.01**
	Ear(s)	0.77	0.58	2.17 (0.70–6.75)	0.18
	Feces	0.06	0.25	1.06 (0.65–1.75)	0.81
	Oronasopharyngeal and respiratory	0.12	0.22	1.13 (0.73–1.73)	0.59
	**Other sites or mixed**	**0.63**	**0.07**	**1.87 (1.62**–**2.15)**	**<0.01**
**Cats**					
Intercept		−3.20	0.24	0.04 (0.03–0.07)	
Sex	Female	–	–	1.00	–
	**Male**	**0.31**	**0.11**	**1.37 (1.11**–**1.68)**	**<0.01**
	Unspecified	0.06	0.30	1.06 (0.59–1.92)	0.84
Genetic breed group^e^	West Europe	–	–	1.00	–
	Asian	0.10	0.29	1.10 (0.62–1.96)	0.74
	Crossbreed	0.11	0.21	1.12 (0.74–1.70)	0.59
	Not yet genetically classified	−0.41	0.37	0.66 (0.32–1.37)	0.27
	Unknown breed	−0.35	0.26	0.71 (0.42–1.18)	0.19
RCVS accreditation	Not accredited	–	–	1.00	–
	Accredited	0.23	0.12	1.25 (0.98–1.60)	0.07
Sampling type/site	Urine	–	–	1.00	–
	Anal region (including anal sacs)	0.27	0.38	1.32 (0.63–2.75)	0.47
	Feces	−1.07	0.60	0.34 (0.11–1.11)	0.07
	**Oronasopharyngeal and respiratory**	**0.67**	**0.25**	**1.95 (1.19**–**3.18)**	**0.01**
	**Other sites or mixed**	**0.65**	**0.17**	**1.91 (1.38**–**2.63)**	**<0.01**

*^a^Standard error.*

*^b^Odds ratio.*

*^c^95% Confidence interval.*

*^d^Vonholdt et al., 2010.*

*^e^Lipinski et al., 2008.*

*For both models, veterinary practice site (dogs, variance = 0.22, standard deviation, *SD*, 0.47; cats, variance = 0.74, *SD* = 0.86) was modeled as a random effect; for dogs, laboratory site (variance = 0.99, *SD* = 1.00) was also included. Significant categories within a variable are emboldened.*

### Laboratory-Based Characterization of Resistant *Enterobacteriaceae* Isolates

#### Referring Laboratories

In total, 146 isolates (130 canine, 16 feline) were submitted for further characterization, including *E. coli* (*n* = 106), *P. mirabilis* (*n* = 12) and *K. pneumoniae* (*n* = 10) (Supplementary Material 4). Of these, 30.8% (CI 22.4–39.2) were indicated as having received antimicrobial therapy prior to sample submission, though 68.4%, (CI 60.2–76.6) were of unknown prior therapy status. Most isolates had been characterized via MIC (67.2% of isolates, CI 58.4–75.9), the remainder via disk diffusion (32.9%, CI 23.7–42.1). CLSI was the most utilized interpretation guideline (67.8%, CI 57.8–77.8), followed by EUCAST (19.8%, CI 9.9–29.7); BSAC (9.6%, CI 4.3–15.0), and CLSI-vet (2.7%, CI 0.0–5.3). 82.8% (CI 76.4–89.3) of isolates were reported by referring laboratories as MDR, and 97.8% (CI 95.4–100.0) were reported as phenotypically resistant to >1 3rd/4th generation cephalosporin, fluoroquinolones or polymyxins. An ESBL phenotype was indicated in 13.7% (CI 7.2–20.1) of submitted isolates, though 60.4% (CI 50.3–70.6) did not report an ESBL test.

#### University of Liverpool

Phenotypic results from our laboratory showed MDR in 83.6% (CI 77.2–90.0) of isolates; 71.3% (CI 63.7–78.9) were resistant to 3rd/4th generation cephalosporins and/or fluoroquinolones. An ESBL-producing phenotype was found in 34.2% (CI 26.3–42.2) of isolates (*n* = 41 *E. coli*; *n* = 5 *K. pneumoniae*; *n* = 2 *P. mirabilis*; *n* = 1 *Proteus* spp.; *n* = 1 *Citrobacter koseri*), whereas a pattern suggestive of AmpC-production was observed in 8.9% (CI 4.2-13.6) of isolates (Supplementary Material 4; *n* = 11 *E. coli*; *n* = 1 *K. pneumoniae*; *n* = 1 *Enterobacter asburiae*).

#### Genomic Analysis

In total, 95 *E. coli* isolates were selected for WGS based on evidence of phenotypic ESBL production (*n* = 38 isolates), MDR (*n* = 68) and/or presence of plasmid-associated AmpC (*n* = 24) or plasmid-associated fluoroquinolone resistance genes (*n* = 6) on PCR (Supplementary Material 4).

**FIGURE 3:**
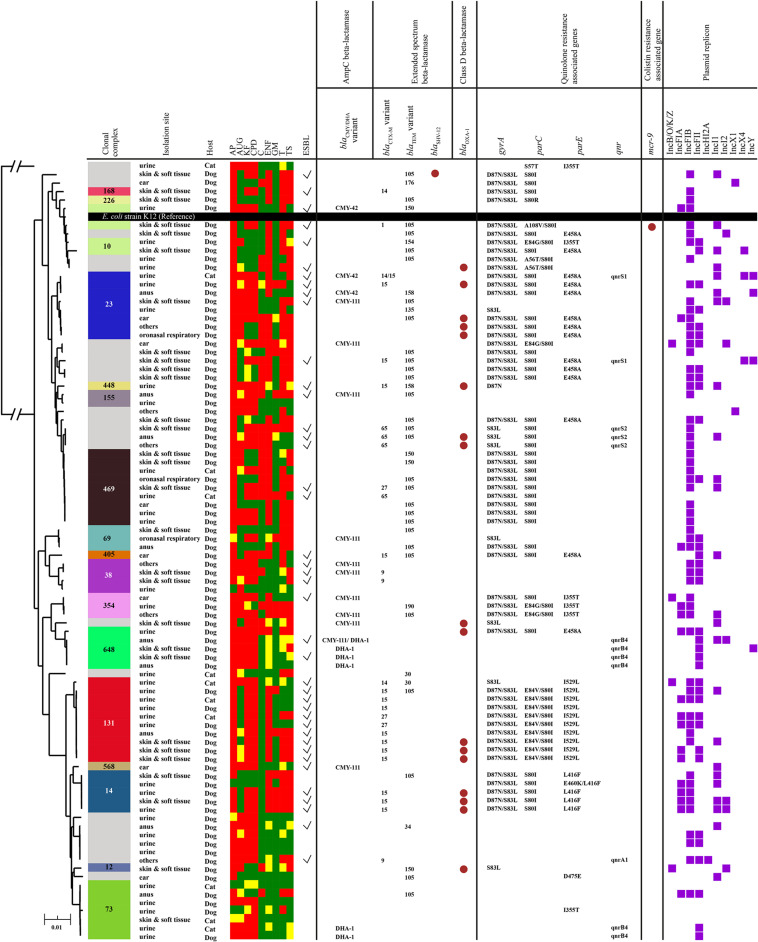
Maximum likelihood phylogenetic tree constructed by core genome SNPs of 91 *E. coli* isolates using the genome of *E. coli* K12 as a reference. Colored bands and numbers in the first column indicate clonal complex (CC) of the isolate (gray color indicates unassigned CC). A heatmap represents antimicrobial susceptibility (red = resistance; yellow = intermediate; green = susceptible). Brown circles in columns six and eleven represent the presence of beta-lactamase genes and the mcr-9 gene, respectively. The seventh – ninth columns represent QRDR mutations in gyrA, parC, and parE, respectively. The tenth column shows the presence/absence of the plasmid-mediated quinolone resistance gene, qnr, in each isolate. Purple squares in the last column indicate plasmid replicons identified in each isolate. Abbreviations for antibiotics tested here are as follows: AP, ampicillin; AUG, clavulanic acid potentiated amoxicillin; KF, cephalothin; CPD, cefpodoxime; C, chloramphenicol; ENF, enrofloxacin; GM, gentamicin; T, tetracycline; TS, trimethoprim potentiated sulfamethoxazole.

Via WGS, MLST results were obtained from 91 *E. coli* isolates, identifying 38 sequence types (ST), most notably ST131 (*n* = 10), ST162 (*n* = 9), ST88, ST372, and ST1193 (*n* = 5 each), ST73 (*n* = 3) and ST69 (*n* = 3). In total, 17 clonal complexes (CC) were identified, most notably CC131 (*n* = 10), CC469 (*n* = 9), CC23 (*n* = 8) and CC73 (*n* = 7) (Figure 3). Core genome-based SNP phylogenetic analysis showed ST clustering, though no clear correlation between animal species or sampling site/type could be elucidated.

An ESBL gene was detected in 28/38 phenotypic ESBL-producers. *bla*_CTX–M_ genes predominated (*n* = 27); *bla*_CTX–M–__15_ was most common (*n* = 15), followed by *bla*_CTX–M–__65_ (*n* = 4), *bla*_CTX–M –__27_ (*n* = 3), *bla*_CTX–M–__14_ (*n* = 3), *bla*_CTX–M–__9_ (*n* = 2), and *bla*_CTX–M–__1_ (*n* = 1). In addition, *bla*_SHV–__12_ (*n* = 1) and the inhibitor resistant *bla*_TEM–__158_ (*n* = 2) and *bla*_TEM–__154_ (*n* = 1) were also detected. All ten ST131 isolates were associated with ESBL genes, with *bla*_CTX–M–__15_ (*n* = 7) most prevalent. Plasmid-associated AmpC genes were detected in 19 *E. coli* isolates, comprising *bla*_CMY–__111_ (*n* = 11), *bla*_DHA–__1_ (*n* = 6), and *bla*_CMY–__42_ (*n* = 3). Of broad spectrum β-lactamases, *bla*_TEM–__105_ was most common (*n* = 29), followed by *bla*_OXA–__1_ (*n* = 17), *bla*_TEM–__150_ (*n* = 4), *bla*_TEM–__135_, *bla*_TEM–__176_ and *bla*_TEM–__190_ (*n* = 1 each). Two inhibitor resistant variants, *bla*_TEM–__30_ (*n* = 2) and *bla*_TEM–__34_ (*n* = 1), were also detected.

Presence of both ESBL and AmpC or inhibitor resistant TEM variant enzymes in the same isolate can produce a phenotypic ‘masking’ effect on double disk ESBL testing, owing to the ability of AmpC to hydrolyze both clavulanic acid-potentiated and non-clavulanic acid potentiated cephalosporins (Jacoby, 2009). This can potentially result in an under-estimate of ESBL prevalence on phenotypic testing, as potentially shown here with non-phenotypic ESBL producers found to be carrying ESBL genes. This was likely due to a masking effect of inhibitor resistant *bla*_TEM–__154_ in the first isolate; *bla*_TEM–__158_ and *bla*_CM__Y–__42_ in the second, and *bla*_CTX–M–__9_ and *bla*_CMY–__111_ in the third isolate. An ESBL gene was not detected in 10 phenotypic ESBL *E. coli* isolates, with isolates carrying plasmid-associated AmpC (*bla*_CMY–__111_, *n* = 3; *bla*_DHA–__1_, *n* = 1) or broad spectrum β-lactamase (*bla*_OXA–__1_, *n* = 1) genes alone, or co-carried with *bla*_CMY–__111_ and *bla*_TEM–__105_ (*n* = 2), *bla*_CMY–__42_ and *bla*_TEM–__150_ (*n* = 1), *bla*_CMY–__111_ and *bla*_DHA–__1_ (*n* = 1), and *bla*_DHA–__1_ and *bla*_TEM–__34_ (*n* = 1), respectively. An ESBL (*bla*_CTX–M–__14_ and *bla*_CTX–M–__15_) and AmpC (*bla*_CMY–__42_) MDR isolate also carried the narrow-spectrum β-lactamase gene *bla*_LAP–__2_.

Mutations in the *gyrA*, *parC*, or *parE* quinolone resistance determining region (QRDR) were found in 62 isolates. Twelve isolates carried plasmid-associated quinolone resistance genes (*qnrA1, n* = 1; *qnrB4*, *n* = 6; *qnrS1*, *n* = 2; *qnrS2, n* = 3), though only five had QRDR mutations, and none were associated with significant fluoroquinolone resistance. More broadly, the overall median number of AMR genes per isolate was 6 (range 0–13). Other prominent AMR genes included *aadA1* (*n* = 23), *strA* (*n* = 34), *strB* (*n* = 35), *sul1* (*n* = 35), *sul2* (*n* = 42), *tetA* (*n* = 25), *tetB* (*n* = 26), *tetR* (*n* = 25), and *dfrA17* (*n* = 30). An ESBL (*bla*_CTX–M–__9_) ST372 MDR isolate also carried a plasmid-mediated colistin resistance gene, *mcr-9* (Supplementary Material 4).

A median of 2 (range 0–4) Inc plasmids per isolate was found, with IncFIB being most numerous (*n* = 59), followed by IncFII (*n* = 35), IncI (*n* = 25) IncFIA (*n* = 14), IncY (*n* = 5), IncR (*n* = 3), IncX4 (*n* = 3), IncX1 (*n* = 2), and IncHI2 (*n* = 1). Though plasmids were widely distributed across STs and resistance genes, nine of ten ST131 isolates possessed F-type plasmids (IncFIB, *n* = 7; IncFII, *n* = 7; IncFIA, *n* = 5). Of *bla*_TEM–__105_ isolates (*n* = 29), 26 possessed IncFIB, and of 11 *bla*_CMY–__111_ isolates, 8 possessed IncFIB. The *mcr-9* positive isolate had three plasmids: IncFIB, IncFII, and IncHI2, with IncH12 plasmids previously reported as associated with *mcr-9* (Kieffer et al., 2019).

## Discussion

Here we have adopted an integrated health informatics and bioinformatics approach to conduct nationally representative surveillance of important clinical AMR trends in companion animals. We have also demonstrated that clinical AMR isolates can be retained by VDLs and further characterized to reveal key AMR insights of one health importance.

Resistance trends in *Enterobacteriaceae* are of critical importance to human health (Tacconelli et al., 2018). Considering the inherent one health nature of AMR (O’Neill, 2016), it is crucial to gain further understanding of AMR in our companion animal populations. Through collating AST results from half of companion animal-treating practice sites in the United Kingdom, with data being broadly comparative with both the estimated companion animal population (Aegerter et al., 2017) and demographic features of the RCVS practice register (RCVS, 2018), we believe these data are nationally representative. However, mixed species practice sites were under-represented in our dataset. Veterinary practitioners employed in different sectors (companion animal, farm etc.) have reported varied levels of support for ASTs (De Briyne et al., 2013), a disparity perhaps reflected in actual AST results here. Such findings can serve as a stimulus for localized resistance trend monitoring, potentially providing a powerful tool and motivator for antimicrobial stewardship.

Antimicrobial classes rarely (or never) prescribed to companion animals, for example carbapenems (Singleton et al., 2017), were only rarely reported as being tested here. Critically important antimicrobials currently only used in medical practice should not be recommended for veterinary use. However, there would be value in routinely reporting resistance trends to such antimicrobials in veterinary isolates for public health purposes, as demonstrated by our findings relating to colistin resistance here. It should also be noted that differences between AST interpretation guidelines (e.g., EUCAST and CLSI) limited our ability to compare laboratories in this study, re-affirming a need for greater harmonization between laboratories. AST data summarized here also does not include an individual animal identifier, hence it was not possible to designate multiple submissions originating from the same animal. To ameliorate this limitation, we would support efforts at increasing integration between practice and laboratory information management systems.

Considering the above, individual class-level resistance prevalence was generally lower than reported in other studies, though MDR prevalence was broadly comparative (Marques et al., 2016). While others have classified intermediate susceptibility results as resistant, potentially increasing resistance prevalence (VMD, 2019), doing so here would not have elevated findings to levels comparative with previous study (for example, re-classifying intermediate susceptibility findings as resistant would have increased *E. coli* 3rd generation cephalosporin resistance prevalence in dogs from 8.4 to 10.6%). Previous surveys have largely utilized data supplied by university-based diagnostic laboratories analyzing referral populations (Hernandez et al., 2014; Marques et al., 2016), and here we found increased odds of resistance in isolates originating from referral-only practice sites for dogs. Antimicrobial use prior to culture has been shown to increase odds of isolating resistant bacteria (Hernandez et al., 2014). It is possible that antibiotic therapy prior to referral might in part explain these findings, suggesting a need to stratify surveillance into first opinion and referral populations, broadly in line with human surveillance (PHE, 2020).

Considering animal-level features, genetic breed group, sex and neuter status were associated with 3rd/4th generation cephalosporin resistance odds variation in both species. These findings potentially reflect anatomical (Hernandez et al., 2014) or behavioral differences (Chhetri et al., 2015) between animals, possibly resulting in increased likelihood of bacterial infection or resistance-selecting empirical antimicrobial prescription in some cases. Indeed, we have recently identified such variation in prescribing practices (Singleton et al., 2020). Regarding sample type/site, the anal region in dogs and, oronasopharyngeal and respiratory samples in cats were associated with increased odds. Though resistant *E. coli* isolated from canine nasal or respiratory sites have been described (Morrissey et al., 2016), it has only rarely been implicated as a causative pathogen in human intensive care settings (de Lastours et al., 2015). In dogs and cats, *Enterobacteriaceae* are considered minor components of oral, nasopharyngeal and respiratory microbiota, and their role as respiratory pathogens remains debatable (Dorn et al., 2017). Similarly, it is difficult to determine whether anal region samples reflect intestinal carriage or clinical infection, though could be considered to pose a public health risk, nonetheless. Several bacterial species usually considered contaminants were also reported within AST data (e.g., *Kluyvera* spp.; *Pantoea* spp. etc.); doing so without thorough evaluation of their association with infection is likely to encourage potentially unnecessary antimicrobial prescription.

There are several *Enterobacteriaceae* resistance trends of critical one health importance, not least ESBL- or AmpC-production, fluoroquinolone resistance, carbapenemase producing *Enterobacteriaceae*, and colistin resistance (MacNair et al., 2018). However, due to the primarily clinical purpose behind AST results analyzed here, some phenotypic tests were only rarely reported. Hence, here we aimed to investigate these isolates to gain further insight into dynamics of resistance development and transmission in the companion animal clinical population, utilizing isolates supplied by multiple independent diagnostic laboratories.

Regarding WGS findings, although ST372 *E. coli* has been previously primarily associated with canine infections and relatively rare resistance (Valat et al., 2020), here we identified an MDR ST372 also possessing *mcr-9*, a recently identified plasmid-associated colistin resistance gene (Carroll et al., 2019). In medical practice, colistin is considered an antimicrobial of ‘last resort,’ and reported emergence of plasmid-mediated colistin resistance in 2015 instigated widespread concern (Liu et al., 2016). Although there is some debate regarding the clinical relevance of *mcr-9* (Tyson et al., 2020) and when it first originated (Elbediwi et al., 2021), our finding represents its first identification in a companion animal, and the first identification of a plasmid-associated colistin resistance gene in companion animals in the United Kingdom. Although companion animals are not prescribed colistin in the United Kingdom, they are relatively frequent recipients of another member of the polymyxin class, polymyxin B (Singleton et al., 2017). Concern has been raised over whether such use might be selecting for colistin resistance (Scott et al., 2019), though this remains to be determined in *Enterobacteriaceae.* Nevertheless, these findings serve to further reinforce the role of companion animals as carriers of resistance mechanisms of public health importance, and the need for surveillance to highlight the emergence of such trends.

A diverse collection of ESBL genes were found in *E. coli* isolates undergoing WGS, with the *bla*_CTX–M_ (1, 9, 14, 15, 27, and 65) family dominating. In people, *bla*_CTX–M–__14_ and *bla*_CTX–M–__15_ are of greatest clinical importance (Canton et al., 2012), whereas agricultural species are more commonly associated with *bla*_CTX–M–__1_ carriage (Abraham et al., 2018). In companion animals, *bla*_CTX–M–__1_ (Wedley et al., 2017), *bla*_CTX–M–__14_ (Sun et al., 2016), and *bla*_CTX–M–__15_ (Timofte et al., 2016) have each been identified. Here *bla*_CTX–M–__15_ was most prevalent; concerningly, the human pandemic clone ST131 predominated, further demonstrating links between people and companion animals for important resistance and pathogenic clones (Zhang, 2016). This clone has been detected in companion animal clinical isolates previously, and is considered a driver of *bla*_CTX–M–__15_ dissemination in human and animal populations (Timofte et al., 2016). F-type Inc plasmids were also frequently identified, this group of plasmids (particularly IncFII) being commonly implicated with global dissemination of *bla*_CTX–M_ (Canton et al., 2012). Inhibitor resistant ESBL genes were also detected at low frequency, the *bla*_TEM–__158_ gene having been previously reported in a companion animal referral hospital in the United Kingdom (Tuerena et al., 2016).

Although less frequently identified than ESBL genes, AmpC genes were also identified in these isolates. Despite *bla*_CMY–__2_ being the most well distributed *bla*_CMY_ allele worldwide (Jacoby, 2009), including in companion animals (Wedley et al., 2011), it was not detected here. Instead, the comparatively recently identified *bla*_CMY–__111_ (Kao et al., 2018), representing the first identification in companion animals, and *bla*_DHA–__1_, this gene only recently reported in European companion animals (Belas et al., 2014), were found to predominate. Although the *bla*_CMY_ gene has many variants (Jacoby, 2009), and as such a measured view of this novel finding should be taken, our results do suggest increased AmpC diversity in companion animals. We also detected ST410 in this population, this ST being previously implicated in human and companion animal infection (Timofte et al., 2016), including harboring a carbapenemase resistance gene in a sample of canine origin (Reynolds et al., 2019).

Regarding treatment of ESBL and/or AmpC-mediated infections in companion animals, the appropriateness of using alternative agents, such as the carbapenems and piperacillin-tazobactam, is a topic of ongoing debate in the medical field due to concerns surrounding developing resistance to these ‘last report’ agents (Tamma and Mathers, 2021). Indeed the prescription of such agents to companion animals is not recommended (BSAVA, 2018), severely limiting treatment options for such cases. Accordingly, whilst emergence of these troubling clinical resistance trends in companion animals is often viewed primarily through the lens of associated public health risk, it should also be remembered that enhanced treatment failure risk may well also carry an increased risk to animal welfare.

Quinolone resistance mechanisms are relatively complex, and consist of interactions between chromosomal mutations and plasmid-mediated genes (Hamed et al., 2018). In this study, chromosomal mutations were commonplace, contrasting with the relative scarcity of plasmid-mediated genes. This finding suggests a greater role of chromosomal mutations in conferring clinical resistance in companion animals, in agreement with previous study (de Jong et al., 2018). Considering AMR genes identified more widely, although many of the AMR genes identified here are commonly associated with plasmid types frequently identified in this study, specific, in-depth investigation was out of the scope of this study. Hence, though unlikely, it is possible that some of the assumed transmissible AMR genes discussed here might be chromosomal in origin.

Although this study was successful in identifying clinical isolates of one health importance, we revealed further evidence of AST method and interpretation variation between VDLs. When confirmatory additional testing was performed at the University of Liverpool, although a similar percentage of isolates were reported as MDR by referring laboratories (82.8%) to that found at Liverpool (83.6%), ESBL testing was only reported for a minority of referring VDLs, potentially systematically under-estimating the impact of ESBLs on companion animal practice, and the potential risk posed to public health. This is likely due notification of ESBL presence being of an arguably greater importance for public health/surveillance than for clinical decision making. Hence, we suggest an urgent need to harmonize standard AST testing procedures, methods, and interpretation more closely, including tests of primary public health/surveillance relevance, if companion animal clinical bacterial isolates are to be used for wide scale surveillance in the future.

## Conclusion

This study demonstrated a method by which clinical AST results could be repurposed for near real-time passive and active AMR clinical surveillance at a nationally representative scale in companion animals, providing insights of importance and use to both veterinary practitioners and public health. Using *Enterobacteriaceae* as an exemplar, we found MDR to be common, with prevalence remaining relatively static between 2016 and 2018. We also conducted a pilot study to determine whether MDR *E. coli* isolates could be efficiently retrieved from VDLs and further characterized at the University of Liverpool, revealing presence of resistance genes and sequence types of importance to human and animal health.

## Data Availability Statement

Data utilized during this study is available upon written request to DS (d.a.singleton@liverpool.ac.uk). Whole genome sequence data is freely available via GenBank (accession numbers available, Supplementary Material 4).

## Author Contributions

NW, AR, GP, P-JN, SD, and FS-V conceived the study. DT, StS, MJ, and LR contributed data and isolates to this project. DS and ShS completed laboratory work. PP, RB, and KB completed or supervised bioinformatic components of this work. DS completed primary analyses and wrote the manuscript under the supervision of NW. All authors contributed to the draft review.

## Conflict of Interest

MJ and LR were employed by the veterinary diagnostic company IDEXX Laboratories Ltd. StS was employed by the veterinary diagnostic company NationWide Laboratories/CAPL Ltd. Although employed by the University of Liverpool, DT held primary responsibility for managing a commercial veterinary diagnostic laboratory within the university. The remaining authors declare that the research was conducted in the absence of any commercial or financial relationships that could be construed as a potential conflict of interest.

## Publisher’s Note

All claims expressed in this article are solely those of the authors and do not necessarily represent those of their affiliated organizations, or those of the publisher, the editors and the reviewers. Any product that may be evaluated in this article, or claim that may be made by its manufacturer, is not guaranteed or endorsed by the publisher.
