# Supplementary material for: Temporal, Spatial, and Genomic Analyses of Enterobacteriaceae Clinical Antimicrobial Resistance in Companion Animals Reveals Phenotypes and Genotypes of One Health Concern
Source: Front Microbiol. 2021 Jul 30;12:700698. doi: 10.3389/fmicb.2021.700698 (PMC8362618; doi:10.3389/fmicb.2021.700698)
Supplement: Supplementary file 1 [file Data_Sheet_1.PDF]

Supplementary material

Supplementary material 1: Antimicrobial classes summarised in the current study.

**Antimicrobial class**	**Antimicrobial agent**
Aminoglycosides	Amikacin, Framycetin, Gentamicin, Neomycin, tobramycin
Amphenicols	Chloramphenicol, Florfenicol
Fluoroquinolones	Ciprofloxacin, Enrofloxacin, Marbofloxacin, Orbifloxacin, Ofloxacin, Pradofloxacin
Nitrofurantoin	Nitrofurantoin
Polymyxins	Polymyxin B
Potentiated sulphonamides	Potentiated sulphonamide
Tetracyclines	Doxycycline, Oxytetracycline, Tetracycline
β-lactams	
Amino-penicillins	*Amoxicillin, Ampicillin, Carbenicillin, Piperacillin, Ticarcillin*
1st/2nd generation cephalosporins	*Cefadroxil, Cefuroxime, Cephalexin*
3rd/4th generation cephalosporins	*Cefovecin, Cefpodoxime, Ceftazidime, Ceftiofur*
Potentiated penicillins	*Clavulanic acid potentiated amoxicillin, Clavulanic acid potentiated ticarcillin*
Carbapenems	*Imipenem, Meropenem*

Supplementary material 2: Antimicrobial discs and associated concentrations used to phenotypically assess resistance and identify phenotypic extended spectrum beta-lacatamase production in *Enterobacteriaceae* isolates collected at the University of Liverpool

ANTIMICROBIAL SUSCEPTIBILITY TEST DISCS

Ampicillin 10µg (amino-penicillin);
Clavulanic acid potentiated amoxicillin 10µg/20µg (potentiated penicillin);
Cephalothin 30µg (1st generation cephalosporin);
Cefpodoxime 10µg (3rd generation cephalosporin);
Imipenem 10µg (carbapenem);
Enrofloxacin 5µg (fluoroquinolone);
Gentamicin 10µg (aminoglycoside);
Chloramphenicol 30µg (amphenicol);
Tetracycline 30µg (tetracycline), and
Trimethoprim potentiated sulfamethoxazole 1.25µg/23.75µg (Potentiated sulphonamide)

EXTENDED SPECTRUM BETA-LACTAMASE COMBINATION DISCS

Cefpodoxime 30µg
Cefpodoxime 30µg + clavulanic acid 10µg
Cefotaxime 30µg
Cefotaxime 30µg + clavulanic acid 10µg
Ceftazidime 30µg
Ceftazidime 30µg + clavulanic acid 10µg

Supplementary material 3: Primers utilised for amplification of genes associated with *E. coli*, the ST131:O25b *E. coli* pandemic clone, and *Enterobacteriaceae* resistance genes associated with ESBL-production; AmpC-production, or quinolone resistance. Table also includes expected product sizes, annealing temperatures and a reference.

Target	Primer	Sequence 5' → 3'	Product size (bp)	Annealing temp. (^{o}C)	Reference
uidA	uidAF	CCAAAAGCCAGACAGAGT	623	58	McDaniels et al. (1996)
	uidaR	GCACAGCACATCAAAGAG			
uspA	uspAF	CCGATACGCTGCCAATCAGT	503	56	Bekal et al. (2003)
	uspAR	ACGCAGACCGTAGGCCAGAT			
**ST131: 025b**	trpAF	GCTACGAATCTCTGTTTGCC	427	65 (multiplex)	Clermont et al. (2009)
	trpAR	GCAACGCGGCCTGGCGGAAG			
	025pabBspeF	TCCAGCAGGTGCTGGATCGT	347		
	025pabBspeR	GCGAAATTTTTCGCCGTACTGT			
***bla*$_{TEM}$**	TSO-TF	CATTTCCGTGTCGCCCTTATTC	800	60 (multiplex)	Dallenne et al. (2010)
	TSO-TR	CGTTCATCCATAGTTGCCTGAC			
***bla*$_{SHV}$**	TSO-SF	AGCCGCTTGAGCAAATTAAAC	713		
	TSO-SR	ATCCCGCAGATAAATCACCAC			
***bla*$_{OXA}$**	TSO-OF	GGCACCAGATTCAACTTTCAAG	564		
	TSO-OR	GACCCCAAGTTTCCTGTAAGTG			
***bla*$_{CTX-M}$**	CTX-MU1	ATGTGCAGYACCAGTAARGTKATGGC	593	58	Boyd et al. (2004)
	CTX-MU2	TGGGTRAARTARGTSACCAGAAYCAGCGG			
***bla*$_{CTX-M}$ Group 1**	CTX-Mgp1F	CCCATGGTTAAAAAATCACTGC	876	55	Carattoli et al. (2008)
	CTX-Mgp1R	CAGCGCTTTTGCCGTCTAAG			
***bla*$_{CTX-M}$ Group 2**	CTX-Mgp2F	ATGATGACTCAGAGCATTCGC	893	55	Hopkins et al. (2006)
	CTX-Mgp2R	TCAGAAACCGTGGGTTACGAT			
***bla*$_{CTX-M}$ Group 9**	CTX-Mgp9F	ATGGTGACAAAGAGAGTGCAAC	876	55	Batchelor et al. (2005)
	CTX-Mgp9R	TTACAGCCCTTCGGCGATG			
***bla*$_{AmpC}$**	CITMf	TGGCCAGAACTGACAGGCAAA	462	64	Perez-Perez and Hanson (2008)
	CITMr	TTTCTCCTGAACGTGGCTGGC			
	DHAMf	AACTTTCACAGGTGTGCTGGGT	405	64 (multiplex)	Perez-Perez and Hanson (2008)
	DHAMr	CCGTACGCATACTGGCTTTGC			
	ACCMf	AACAGCCTCAGCAGCCGGTTA	346		
	ACCMr	TTCGCCGCAATCATCCCTAGC			
	EBCMf	TCGGTAAAGCCGATGTTGCGG	302		
	EBCMr	CTTCCACTGCGGCTGCCAGTT			
	FOXMf	AACATGGGGTATCAGGGAGATG	190		
	FOXMr	CAAAGCGCGTAACCGGATTGG			
	MOXMF	GCTGCTCAAGGAGCACAGGAT	520	64	Perez-Perez and Hanson (2008)
	MOXMR	CACATTGACATAGGTGTGGTGC			
qnrA	qnrAF	ATTTCTCACGCCAGGATTTG	516	53 (multiplex)	Robicsek et al. (2006)
	qnrAR	GATCGGCAAAGGTTAGGTCA			
qnrB	qnrBF	GATCGTGAAAGCCAGAAAGG	469		
	qnrBR	ACGATGCCTGGTAGTTGTTGTCC			
qnrS	qnrSF	ACGACATTCGTCAACTGCAA	417		
	qnrSR	TAAATTGGCACCCTGTAGGC			
aac(6')-Ib	aac(6')-Ib-F	TTGCGATGCTCTATGATGGGCTA	420	55	Park et al. (2006)
	aac(6')-Ib-R	CTCGAATGCCTGGCGTGTTT			

Supplementary material 4: Phenotypic and genotypic (polymerase chain reaction, PCR, and whole genome sequencing, WGS) findings for *Enterobacteriaceae* canine and feline clinical isolates (*n*=148) which were identified by veterinary diagnostic laboratories (*n*=3) as being multi-drug resistant, extended spectrum β-lactamase (ESBL) producing, and / or individually resistant to 3rd/4th generation cephalosporins, fluoroquinolones and / or polymyxins.

Animal species	Sample type/site	Referring laboratory reported bacterial species	Confirmed E. coli	ESBL phenotype	Resistance phenotype	PCR ST131:O25b	PCR plasmid-mediated AMR genes	GenBank accession number	WGS Sequence type	WGS Clonal complex	WGS plasmid-mediated AMR genes
Canine	Other / mixed	*Escherichia coli*	1	1	AMP KF CPD ENF TET POTS	0	bla_{TEM} $bla_{\text{CTX-M-1}}$	SAMN16278389	10	10	$AGly_{\text{aadA5}}$ $AGly_{\text{strA}}$ $AGly_{\text{strB}}$ $bla_{\text{CTX-M-1}}$ $bla_{\text{TEM-105}}$ Sul_{sul1} Sul_{sul2} Tet_{tetA} Tet_{tetB} Tet_{tetR} Tmt_{tetR}
Canine	Urine	*Escherichia coli*	1	1	AMP AUG KF CPD TET	0	bla_{TEM} bla_{CIT}	SAMN16279754	10	10	$AGly_{\text{aadA1-pm}}$ $bla_{\text{CMY-42}}$ $bla_{\text{TEM-150}}$ Sul_{sul1} Tet_{tetA} Tet_{tetR}
Canine	Urine	*Escherichia coli*	1	0	AMP AUG ENF TET	0	bla_{TEM}	SAMN16279757	10	10	$AGly_{\text{aadA1-pm}}$ $AGly_{\text{sat-2A}}$ $bla_{\text{TEM-154}}$ Tet_{tetB} Tmt_{dfrA1}
Canine	Other / mixed	*Escherichia coli*	1	0	AMP AUG C POTS	0	bla_{TEM} bla_{OXA} *aac(6')-Ib*	SAMN16278410	12	12	$AGly_{\text{aadA1-pm}}$ $AGly_{\text{strA}}$ $bla_{\text{TEM-135}}$ $bla_{\text{TEM-135}}$ Phe_{floR} Sul_{sul1} Sul_{sul2}
Canine	Other / mixed	*Escherichia coli*	1	1	AMP KF CPD POTS	0	bla_{OXA}	SAMN16279733	38	38	$AGly_{\text{aadA1-pm}}$ $AGly_{\text{aph3-Ia}}$ $AGly_{\text{sat-2A}}$ $AGly_{\text{strA}}$ $AGly_{\text{strB}}$ $bla_{\text{CTX-M}}$-9 Sul_{sul2} Tmt_{dfrA1}
Canine	Urine	*Escherichia coli*	1	0	AUG	0		SAMN16279764	38	38	$bla_{\text{DHA-1}}$
Canine	Anal region	*Escherichia coli*	1	1	AMP KF CPD C GM TET POTS	0	bla_{TEM} bla_{CIT}	SAMN16278403	58	155	$AGly_{\text{aac3-IId}}$ $AGly_{\text{strA}}$ $AGly_{\text{strB}}$ $bla_{\text{CMY-111}}$ Phe_{floR} Sul_{sul2} Tmt_{dfrA14}
Canine	Other / mixed	*Escherichia coli*	1	0	AMP TET POTS	0	bla_{TEM}	SAMN16278398	69	69	$AGly_{\text{aadA1-pm}}$ $AGly_{\text{strA}}$ $AGly_{\text{strB}}$ Sul_{sul1} Sul_{sul2} Tet_{tetA} Tet_{tetR} Tmt_{dhfr7}
Canine	Anal region	*Escherichia coli*	1	0	AMP ENF TET POTS	0	bla_{TEM}	SAMN16279743	69	69	$AGly_{\text{strA}}$ $AGly_{\text{strB}}$ $bla_{\text{OXA-1}}$ Sul_{sul2} Tet_{tetB} Tmt_{dfrA14}
Canine	Oronasal / respiratory	*Escherichia coli*	1	0	KF CPD GM TET POTS	0	bla_{CIT}	SAMN16279756	69	69	$AGly_{\text{aac3-IId}}$ $AGly_{\text{aadA5}}$ $bla_{\text{CMY-111}}$ Sul_{sul1} Tet_{tetB} Tmt_{tetR}
Canine	Urine	*Escherichia coli*	0	0	CPD TET	0	bla_{TEM} bla_{CIT} *qnrS*	SAMN16278411	73	73	$bla_{\text{OXA-1}}$
Canine	Anal region	*Escherichia coli*	1	0	AMP C TET POTS	0	bla_{TEM} bla_{OXA} $bla_{\text{CTX-M}}$	SAMN16279742	73	73	$AGly_{\text{aadA1-pm}}$ $AGly_{\text{strA}}$ $AGly_{\text{strB}}$ $bla_{\text{TEM-105}}$ Phe_{catA1} Sul_{sul1} Sul_{sul2} Tet_{tetB} Tmt_{dfrA1}
Feline	Urine	*Escherichia coli*	1	0		0		SAMN16279762	73	73	
Canine	Urine	*Escherichia coli*	1	0	AMP AUG KF CPD	0		SAMN16279766	73	73	$bla_{\text{TEM-105}}$
Canine	Other / mixed	*Escherichia coli*	1	0	AMP AUG KF CPD TET	0		SAMN16279740	75	Unknown	$Fcyn_{\text{FosA7}}$ Tet_{tetA} Tet_{tetR}
Canine	Ear	*Escherichia coli*	1	1	AMP AUG KF CPD	0	bla_{CIT}	SAMN16278409	80	568	$bla_{\text{CMY-111}}$ $bla_{\text{OXA-1}}$
Canine	Other / mixed	*Escherichia coli*	1	1	AMP AUG KF CPD TET POTS	0	bla_{TEM} bla_{CIT}	SAMN16278423	88	23	$AGly_{\text{aadA1-pm}}$ $AGly_{\text{sat-2A}}$ $AGly_{\text{strA}}$ $AGly_{\text{strB}}$ $bla_{\text{CMY-111}}$ $bla_{\text{TEM-105}}$ Phe_{catA1} Sul_{sul2} Tet_{tetB} Tmt_{dfrA1}
Canine	Oronasal / respiratory	*Escherichia coli*	1	0	AMP AUG C ENF TET POTS	0	bla_{OXA}	SAMN16279744	88	23	$AGly_{\text{aadA1-pm}}$ $AGly_{\text{aph3-Ia}}$ $bla_{\text{OXA-1}}$ Phe_{catA1} Sul_{sul1} Tet_{tetB} Tmt_{dfrA5}
Canine	Other / mixed	*Escherichia coli*	1	0	AMP AUG C ENF TET POTS	0	bla_{OXA}	SAMN16279745	88	23	$AGly_{\text{aadA1-pm}}$ $AGly_{\text{aph3-Ia}}$ $bla_{\text{OXA-1}}$ $bla_{\text{TEM-105}}$ Phe_{catA1} Sul_{sul1} Tet_{tetB} Tmt_{dfrA5}
Canine	Ear	*Escherichia coli*	1	0	AMP AUG KF CPD C ENF TET POTS	0	bla_{TEM} bla_{OXA} *aac(6')-Ib*	SAMN16279746	88	23	$AGly_{\text{aadA1-pm}}$ $AGly_{\text{aph3-Ia}}$ $AGly_{\text{strA}}$ $AGly_{\text{strB}}$ $bla_{\text{DHA-1}}$ Phe_{floR} Sul_{sul1} Sul_{sul2}
Canine	Urine	*Escherichia coli*	1	0	AMP AUG KF CPD TET	0	bla_{TEM}	SAMN16279753	88	23	$bla_{\text{TEM-135}}$ $bla_{\text{TEM-135}}$ Tet_{tetA} Tet_{tetR}

Animal species	Sample type/site	Referring laboratory reported bacterial species	Confirmed E. coli	ESBL phenotype	Resistance phenotype	PCR ST131:O25b	PCR plasmid-mediated AMR genes	GenBank accession number	WGS Sequence type	WGS Clonal complex	WGS plasmid-mediated AMR genes
Canine	Other / mixed	*Escherichia coli*	1	1	AMP KF CPD ENF TET POTS	0	bla_{OXA}	SAMN16278433	93	168	$AGly_{\text{aadA1-pm}}$ $AGly_{\text{strA}}$ $AGly_{\text{strB}}$ $bla_{\text{CTX-M-14}}$ Sul_{sul1} Sul_{sul2} Tet_{tetA} Tet_{tetR} Tmt_{dfrA1}
Canine	Urine	*Escherichia coli*	1	0	AUG KF CPD	0		SAMN16279763	127	Unknown	
Canine	Other / mixed	*Escherichia coli*	1	1	AMP KF CPD ENF TET POTS	1	bla_{OXA} $bla_{\text{CTX-M-15}}$ *aac(6')-Ib*	SAMN16278387	131	131	$AGly_{\text{aadA5}}$ $bla_{\text{CTX-M-15}}$ $bla_{\text{TEM-105}}$ Sul_{sul1} Tet_{tetA} Tet_{tetR} Tmt_{tetR}
Feline	Urine	*Escherichia coli*	1	1	AMP AUG KF CPD	0	bla_{TEM} bla_{OXA}	SAMN16278392	131	131	$bla_{\text{CTX-M-14}}$ $bla_{\text{TEM-105}}$ $bla_{\text{TEM-30}}$
Canine	Anal region	*Escherichia coli*	1	1	AMP KF CPD ENF GM TET POTS	1	bla_{OXA} $bla_{\text{CTX-M-15}}$ *aac(6')-Ib*	SAMN16278395	131	131	$AGly_{\text{aac3-IIa}}$ $AGly_{\text{aadA5}}$ $bla_{\text{CTX-M-15}}$ $bla_{\text{OXA-1}}$ Sul_{sul1} Tet_{tetA} Tet_{tetR} Tmt_{tetR}
Canine	Other / mixed	*Escherichia coli*	1	1	AMP KF CPD ENF TET POTS	0	bla_{OXA} $bla_{\text{CTX-M-15}}$ *aac(6')-Ib*	SAMN16278396	131	131	$AGly_{\text{aadA5}}$ $bla_{\text{CTX-M-15}}$ $bla_{\text{TEM-105}}$ Sul_{sul1} Tet_{tetA} Tet_{tetR} Tmt_{tetR}
Canine	Urine	*Escherichia coli*	1	1	AMP KF CPD ENF GM	1	bla_{TEM} $bla_{\text{CTX-M-15}}$	SAMN16278407	131	131	$AGly_{\text{aac3-IId}}$ $bla_{\text{CTX-M-15}}$
Canine	Other / mixed	*Escherichia coli*	1	1	AMP KF CPD ENF TET POTS	0	bla_{OXA} $bla_{\text{CTX-M-15}}$ *aac(6')-Ib*	SAMN16278428	131	131	$AGly_{\text{aadA5}}$ $bla_{\text{CTX-M-15}}$ $bla_{\text{OXA-1}}$ Sul_{sul1} Tet_{tetA} Tet_{tetR} Tmt_{tetR}
Feline	Urine	*Escherichia coli*	1	1	AMP KF CPD ENF	1	$bla_{\text{CTX-M-15}}$	SAMN16278431	131	131	$bla_{\text{CTX-M-15}}$ $bla_{\text{TEM-105}}$
Canine	Urine	*Escherichia coli*	1	1	AMP KF CPD ENF	1	bla_{OXA}	SAMN16279736	131	131	$bla_{\text{CTX-M-27}}$ $bla_{\text{OXA-1}}$
Canine	Urine	*Escherichia coli*	1	1	AMP KF CPD ENF	0	$bla_{\text{CTX-M-15}}$	SAMN16279755	131	131	$bla_{\text{CTX-M-15}}$
Feline	Urine	*Escherichia coli*	1	1	AMP KF CPD ENF TET POTS	1	bla_{OXA}	SAMN16279770	131	131	$AGly_{\text{aadA5}}$ $AGly_{\text{strA}}$ $AGly_{\text{strB}}$ $bla_{\text{CTX-M-27}}$ $bla_{\text{OXA-1}}$ Sul_{sul1} Sul_{sul2} Tet_{tetA} Tet_{tetR} Tmt_{tetR}
Feline	Urine	*Escherichia coli*	1	0	AMP AUG KF CPD	0		SAMN16278405	133	Unknown	
Canine	Urine	*Escherichia coli*	1	0	AMP AUG KF CPD	0		SAMN16278413	155	155	$bla_{\text{TEM-105}}$
Canine	Urine	*Escherichia coli*	1	0	AMP AUG KF CPD ENF TET POTS	0	bla_{TEM}	SAMN16278385	162	469	$AGly_{\text{aadA5}}$ $AGly_{\text{strA}}$ $AGly_{\text{strB}}$ $bla_{\text{TEM-105}}$ Sul_{sul2} Tet_{tetB} Tmt_{tetR}
Canine	Urine	*Escherichia coli*	1	0	AMP AUG KF CPD ENF TET POTS	0	bla_{TEM}	SAMN16278386	162	469	$AGly_{\text{aadA5}}$ $AGly_{\text{strA}}$ $AGly_{\text{strB}}$ $bla_{\text{OXA-1}}$ Sul_{sul2} Tet_{tetB} Tmt_{tetR}
Canine	Ear	*Escherichia coli*	1	0	AMP ENF TET POTS	0	bla_{TEM}	SAMN16278424	162	469	$AGly_{\text{aadA5}}$ $AGly_{\text{strA}}$ $AGly_{\text{strB}}$ $bla_{\text{TEM-105}}$ Sul_{sul2} Tet_{tetB} Tmt_{tetR}
Feline	Urine	*Escherichia coli*	1	1	AMP KF CPD C ENF GM TET	0	bla_{OXA}	SAMN16279741	162	469	$AGly_{\text{aac-IVa}}$ $AGly_{\text{aph4-Ia}}$ $bla_{\text{CTX-M-65}}$ $bla_{\text{TEM-105}}$ Phe_{floR} Sul_{sul2}
Canine	Other / mixed	*Escherichia coli*	1	1	AMP KF CPD C ENF TET POTS	0	bla_{TEM} bla_{OXA}	SAMN16279751	162	469	$AGly_{\text{strA}}$ $AGly_{\text{strB}}$ $bla_{\text{CTX-M-27}}$ Phe_{catA2} Sul_{sul2} Tet_{tetA} Tet_{tetR} Tmt_{dfrA14}
Feline	Urine	*Escherichia coli*	1	0	AMP KF CPD C ENF TET	0		SAMN16279752	162	469	$AGly_{\text{strA}}$ $AGly_{\text{strB}}$ Phe_{floR} Sul_{sul2} Tet_{tetA} Tet_{tetR}
Canine	Other / mixed	*Escherichia coli*	1	0	AMP AUG C ENF POTS	0	bla_{TEM} *aac(6')-Ib*	SAMN16279758	162	469	$AGly_{\text{aadA1-pm}}$ $AGly_{\text{aadA5}}$ $AGly_{\text{strA}}$ $AGly_{\text{strB}}$ $bla_{\text{TEM-105}}$ $bla_{\text{TEM-150}}$ Phe_{floR} Sul_{sul1} Sul_{sul2} Tmt_{dfrA1} Tmt_{tetR}
Canine	Oronasal / respiratory	*Escherichia coli*	1	0	AMP C ENF TET POTS	0	bla_{TEM}	SAMN16279768	162	469	$AGly_{\text{aadA5}}$ $AGly_{\text{strA}}$ $AGly_{\text{strB}}$ Phe_{floR} Sul_{sul2} Tet_{tetB} Tmt_{tetR}
Canine	Other / mixed	*Escherichia coli*	1	0	AMP AUG C ENF POTS	0	bla_{TEM}	SAMN16279772	162	469	$AGly_{\text{aadA1-pm}}$ $AGly_{\text{aadA5}}$ $AGly_{\text{strA}}$ $AGly_{\text{strB}}$ $bla_{\text{OXA-1}}$ $bla_{\text{TEM-150}}$ Phe_{floR} Sul_{sul1} Sul_{sul2} Tmt_{dfrA1} Tmt_{tetR}
Feline	Urine	*Escherichia coli*	1	0	AMP TET POTS	0	bla_{TEM}	SAMN16279735	174	Unknown	$AGly_{\text{strA}}$ $AGly_{\text{strB}}$ $bla_{\text{TEM-30}}$ Sul_{sul1} Tet_{tetA} Tet_{tetR} Tmt_{dfrA5}
Canine	Other / mixed	*Escherichia coli*	1	0	AMP ENF TET POTS	0	bla_{TEM}	SAMN16278416	226	226	$AGly_{\text{aadA5}}$ $AGly_{\text{aph3-Ia}}$ $AGly_{\text{strA}}$ $AGly_{\text{strB}}$ Sul_{sul2} Tet_{tetB} Tmt_{tetR}

Animal species	Sample type/site	Referring laboratory reported bacterial species	Confirmed E. coli	ESBL phenotype	Resistance phenotype	PCR ST131:O25b	PCR plasmid-mediated AMR genes	GenBank accession number	WGS Sequence type	WGS Clonal complex	WGS plasmid-mediated AMR genes
Canine	Other / mixed	*Escherichia coli*	1	0	AMP AUG KF CPD ENF GM TET POTS	0	bla_{TEM} bla_{CIT}	SAMN16278415	354	354	$AGly_{\text{aac3-IId}}$ $AGly_{\text{strA}}$ $AGly_{\text{strB}}$ $bla_{\text{CMY-111}}$ $bla_{\text{TEM-105}}$ Sul_{sul2} Tet_{tetB} Tmt_{tetR}
Canine	Urine	*Escherichia coli*	1	0	AMP AUG C ENF GM TET POTS	0	bla_{TEM}	SAMN16278430	354	354	$AGly_{\text{aac3-IId}}$ $AGly_{\text{aadA1-pm}}$ $bla_{\text{TEM-190}}$ Phe_{floR} Sul_{sul1} Sul_{sul2} Tet_{tetB} Tmt_{dfrA1} Tmt_{tetR}
Canine	Ear	*Escherichia coli*	1	1	AMP AUG KF CPD ENF	0	bla_{CIT}	SAMN16279730	354	354	$bla_{\text{CMY-111}}$ $bla_{\text{DHA-1}}$
Canine	Other / mixed	*Escherichia coli*	1	1	AMP AUG KF CPD TET POTS	0	bla_{OXA} bla_{DHA} *qnrA aac(6')-Ib*	SAMN16278399	372	Unknown	$AGly_{\text{aadA2}}$ $AGly_{\text{aadB}}$ $bla_{\text{CTX-M-9}}$ $bla_{\text{TEM-105}}$ *mcr-901* $\text{Flq}_{\text{qnr-A1}}$ Sul_{sul1} Tet_{tetA} Tet_{tetR}
Canine	Anal region	*Escherichia coli*	1	1	AMP AUG KF CPD	0	bla_{DHA}	SAMN16278402	372	Unknown	$bla_{\text{TEM-105}}$ bla_{TEM}-34 Sul_{sul2}
Canine	Urine	*Escherichia coli*	1	0	AMP AUG KF CPD	0		SAMN16278408	372	Unknown	
Canine	Urine	*Escherichia coli*	1	0	AMP AUG KF CPD	0		SAMN16278418	372	Unknown	
Canine	Urine	*Escherichia coli*	1	0	AMP KF CPD	0		SAMN16278419	372	Unknown	$bla_{\text{DHA-1}}$
Canine	Ear	*Escherichia coli*	1	1	AMP KF CPD C ENF TET	0	bla_{TEM} $bla_{\text{CTX-M-15}}$	SAMN16279767	405	405	$AGly_{\text{strB}}$ $bla_{\text{CTX-M-15}}$ $bla_{\text{TEM-105}}$ $Fcyn_{\text{FosA4}}$ Phe_{floR} Tet_{tetA} Tet_{tetR}
Feline	Urine	*Escherichia coli*	1	1	AMP AUG KF CPD ENF POTS	0	bla_{OXA} $bla_{\text{CTX-M-15}}$ bla_{CIT} *qnrS aac(6')-Ib*	SAMN16278427	410	23	$AGly_{\text{strA}}$ $AGly_{\text{strB}}$ $bla_{\text{CMY-42}}$ $bla_{\text{CTX-M-14}}$ $bla_{\text{CTX-M-15}}$ $bla_{\text{OXA-1}}$ $Flq_{\text{qnr-S1}}$ Sul_{sul2} Tmt_{dfrA14}
Canine	Anal region	*Escherichia coli*	1	0	AMP AUG KF CPD ENF	0	bla_{TEM} bla_{CIT} *aac(6')-Ib*	SAMN16279750	410	23	$bla_{\text{CMY-42}}$ $bla_{\text{TEM-105}}$ $bla_{\text{TEM-158}}$
Canine	Urine	*Escherichia coli*	1	1	AMP KF CPD ENF GM TET	0	bla_{OXA} $bla_{\text{CTX-M-15}}$	SAMN16279774	410	23	$AGly_{\text{aac3-IIa}}$ $bla_{\text{CTX-M-15}}$ *bla*LAP-2 $bla_{\text{SHV-12}}$ Tet_{tetA} Tet_{tetR}
Canine	Other / mixed	*Escherichia coli*	1	0	AMP ENF GM TET POTS	0	bla_{TEM}	SAMN16278388	533	Unknown	$AGly_{\text{aac3-IId}}$ $AGly_{\text{aadA5}}$ $AGly_{\text{strA}}$ $AGly_{\text{strB}}$ $bla_{\text{TEM-105}}$ Sul_{sul1} Sul_{sul2} Tet_{tetA} Tet_{tetR} Tmt_{tetR}
Canine	Ear	*Escherichia coli*	1	0	AMP KF CPD C ENF GM POTS	0	bla_{CIT}	SAMN16279738	533	Unknown	$AGly_{\text{aadA1-pm}}$ $AGly_{\text{aadB}}$ $bla_{\text{CMY-111}}$ $bla_{\text{OXA-1}}$ Phe_{floR} Sul_{sul1} Sul_{sul2}
Canine	Other / mixed	*Escherichia coli*	1	0	AMP ENF TET POTS	0	bla_{TEM}	SAMN16278432	617	10	$AGly_{\text{aadA2}}$ $AGly_{\text{aadA5}}$ Sul_{sul1} Tet_{tetB} Tmt_{tetR}
Canine	Urine	*Escherichia coli*	1	0	AMP C ENF POTS	0	bla_{TEM}	SAMN16278394	744	Unknown	$AGly_{\text{aadA1-pm}}$ $AGly_{\text{strA}}$ $AGly_{\text{strB}}$ Phe_{catA1} Sul_{sul1} Sul_{sul2} Tmt_{dfrA1}
Canine	Urine	*Escherichia coli*	1	1	AMP C ENF TET POTS	0	bla_{OXA}	SAMN16278412	744	Unknown	$AGly_{\text{aadA1-pm}}$ Phe_{floR} Sul_{sul1} Sul_{sul2} Tet_{tetB}
Canine	Ear	*Escherichia coli*	1	0	AMP	0	bla_{TEM}	SAMN16278414	929	Unknown	$bla_{\text{TEM-105}}$
Canine	Urine	*Escherichia coli*	1	1	AMP AUG KF CPD C TET	0	bla_{TEM} bla_{OXA} $bla_{\text{CTX-M-15}}$	SAMN16279773	940	448	$AGly_{\text{aadA1-pm}}$ $AGly_{\text{sat-2A}}$ $bla_{\text{CTX-M-15}}$ $bla_{\text{OXA-1}}$ $bla_{\text{TEM-158}}$ Phe_{catA1} Tet_{tetB} Tmt_{dfrA1}
Canine	Other / mixed	*Escherichia coli*	1	1	AMP AUG KF CPD TET	0	bla_{CIT}	SAMN16278406	963	38	$AGly_{\text{aph3-Ia}}$ $AGly_{\text{strB}}$ $bla_{\text{CMY-111}}$ $bla_{\text{TEM-105}}$ Sul_{sul2} Tet_{tetB}
Canine	Other / mixed	*Escherichia coli*	1	0	AMP AUG KF CPD C POTS	0	bla_{OXA} bla_{CIT}	SAMN16279734	963	38	$bla_{\text{CMY-111}}$ $bla_{\text{CTX-M-9}}$ Phe_{floR} Sul_{sul2}
Feline	Urine	*Escherichia coli*	1	0	AMP AUG KF CPD	0	bla_{DHA}	SAMN16278420	968	73	$bla_{\text{DHA-1}}$ Flq_{qnrB4} Sul_{sul1} Tmt_{tetR}
Canine	Urine	*Escherichia coli*	1	0	AMP AUG KF CPD	0	bla_{DHA} *aac(6')-Ib*	SAMN16278421	968	73	$bla_{\text{OXA-1}}$ Flq_{qnrB4} Sul_{sul1} Tmt_{tetR}
Canine	Other / mixed	*Escherichia coli*	1	1	AMP KF CPD ENF TET POTS	0	bla_{TEM} bla_{SHV}	SAMN16279749	1011	Unknown	$AGly_{\text{aadA1-pm}}$ $AGly_{\text{strA}}$ $AGly_{\text{strB}}$ Sul_{sul1} Sul_{sul2} Tet_{tetA} Tet_{tetR} Tmt_{dfrA1}
Canine	Other / mixed	*Escherichia coli*	1	0	AMP ENF GM TET POTS	0	bla_{TEM}	SAMN16278391	1193	14	$AGly_{\text{aac3-IId}}$ $AGly_{\text{strA}}$ $AGly_{\text{strB}}$ Sul_{sul2} Tet_{tetB} Tmt_{tetR}

Animal species	Sample type/site	Referring laboratory reported bacterial species	Confirmed E. coli	ESBL phenotype	Resistance phenotype	PCR ST131:O25b	PCR plasmid-mediated AMR genes	GenBank accession number	WGS Sequence type	WGS Clonal complex	WGS plasmid-mediated AMR genes
Canine	Urine	*Escherichia coli*	1	1	AMP KF CPD ENF GM TET POTS	0	bla_{OXA} $bla_{\text{CTX-M-15}}$ *qnrS* *aac(6')-Ib*	SAMN16278422	1193	14	$AGly_{\text{aac3-IIa}}$ $AGly_{\text{strA}}$ $AGly_{\text{strB}}$ $bla_{\text{CTX-M-15}}$ $bla_{\text{TEM-105}}$ Sul_{sul2} Tet_{tetB} Tmt_{tetR}
Canine	Urine	*Escherichia coli*	1	1	AMP KF CPD ENF GM TET POTS	0	bla_{OXA} $bla_{\text{CTX-M-15}}$ *aac(6')-Ib*	SAMN16279739	1193	14	$AGly_{\text{aac3-IIa}}$ $AGly_{\text{strA}}$ $AGly_{\text{strB}}$ $bla_{\text{CTX-M-15}}$ Sul_{sul2} Tet_{tetB} Tmt_{tetR}
Canine	Urine	*Escherichia coli*	1	0	ENF TET POTS	0		SAMN16279761	1193	14	$AGly_{\text{strA}}$ $AGly_{\text{strB}}$ Sul_{sul2} Tet_{tetB} Tmt_{tetR}
Canine	Other / mixed	*Escherichia coli*	1	1	AMP KF CPD ENF GM TET POTS	0	bla_{OXA} $bla_{\text{CTX-M-15}}$ *aac(6')-Ib*	SAMN16279771	1193	14	$AGly_{\text{aac3-IIa}}$ $AGly_{\text{strA}}$ $AGly_{\text{strB}}$ $bla_{\text{CTX-M-15}}$ Sul_{sul2} Tet_{tetB} Tmt_{tetR}
Canine	Other / mixed	*Escherichia coli*	1	0	AMP C ENF TET POTS	0	bla_{TEM}	SAMN16278393	1196	Unknown	$AGly_{\text{aadA1-pm}}$ $AGly_{\text{aadA2}}$ $bla_{\text{TEM-105}}$ Phe_{cmlA1} Sul_{sul3} Tet_{tetA} Tet_{tetR} Tmt_{dfrA1}2 *qacH*
Canine	Other / mixed	*Escherichia coli*	1	0	AMP C ENF TET POTS	0	bla_{TEM}	SAMN16279732	1196	Unknown	$AGly_{\text{aadA1-pm}}$ $AGly_{\text{aadA2}}$ Phe_{cmlA1} Sul_{sul3} Tet_{tetA} Tet_{tetR} Tmt_{dfrA12} *qacH*
Feline	Other / mixed	*Escherichia coli*	1	0	KF CPD	0		SAMN16279760	1262	73	
Canine	Other / mixed	*Escherichia coli*	1	0	AMP ENF TET POTS	0	bla_{TEM} *aac(6')-Ib*	SAMN16278400	1431	Unknown	$AGly_{\text{strA}}$ $AGly_{\text{strB}}$ $bla_{\text{OXA-1}}$ $bla_{\text{TEM-105}}$ Sul_{sul2} Tet_{tetA} Tet_{tetR} Tmt_{dfrA5}
Canine	Other / mixed	*Escherichia coli*	1	1	AMP KF CPD C ENF TET POTS	0	bla_{TEM} $bla_{\text{CTX-M-15}}$ *qnrS*	SAMN16279759	2006	Unknown	$AGly_{\text{aadA2}}$ $AGly_{\text{strA}}$ $AGly_{\text{strB}}$ $bla_{\text{CTX-M-15}}$ $Flq_{\text{qnr-S1}}$ Phe_{cmlA1} Sul_{sul2} Sul_{sul3} Tet_{tetA} Tet_{tetR} Tmt_{dfrA12} *qacH*
Canine	Anal region	*Escherichia coli*	1	1	AMP KF CPD C ENF	0	bla_{TEM} bla_{OXA} bla_{OXA} *aac(6')-Ib*	SAMN16278401	2179	Unknown	$bla_{\text{CTX-M-65}}$ Flq_{qnrS2} Phe_{catB3} Phe_{floR}
Canine	Other / mixed	*Escherichia coli*	1	1	AMP KF CPD C ENF GM TET POTS	0	bla_{TEM} bla_{OXA} bla_{OXA} *aac(6')-Ib*	SAMN16278425	2179	Unknown	$AGly_{\text{aac3-IId}}$ $AGly_{\text{aadA5}}$ $AGly_{\text{strA}}$ $AGly_{\text{strB}}$ $bla_{\text{CTX-M-65}}$ $bla_{\text{DHA-1}}$ Flq_{qnrS2} Phe_{catB3} Phe_{floR} Sul_{sul1} Sul_{sul2} Tet_{tetA} Tet_{tetR}
Canine	Other / mixed	*Escherichia coli*	1	1	AMP AUG KF CPD C ENF	0	bla_{OXA} bla_{OXA} *aac(6')-Ib*	SAMN16279737	2179	Unknown	$bla_{\text{CTX-M-65}}$ Flq_{qnrS2} Phe_{catB3} Phe_{floR}
Canine	Ear	*Escherichia coli*	1	0	AMP C ENF TET POTS	0	bla_{TEM} *aac(6')-Ib*	SAMN16278384	3014	Unknown	$AGly_{\text{aadA1-pm}}$ $AGly_{\text{aadA2}}$ $AGly_{\text{aph(3'')-Ia}}$ $bla_{\text{TEM-105}}$ $bla_{\text{TEM-176}}$ Phe_{cmlA1} Sul_{sul3} Tet_{tetA} Tet_{tetR} Tmt_{dfr16} *qacH*
Canine	Other / mixed	*Escherichia coli*	1	0	AMP ENF TET	0	bla_{TEM} *aac(6')-Ib*	SAMN16278397	5761	Unknown	$bla_{\text{TEM-105}}$ Tet_{tetB}
Canine	Anal region	*Escherichia coli*	1	0	AMP AUG KF CPD	0	bla_{DHA}	SAMN16278426	5879	648	$bla_{\text{LAP-2}}$ Flq_{qnrB4} Sul_{sul1} Tmt_{tetR}
Canine	Other / mixed	*Escherichia coli*	1	1	AMP AUG KF CPD	0	bla_{DHA}	SAMN16279731	5879	648	$bla_{\text{TEM-105}}$ Flq_{qnrB4} Sul_{sul1} Tmt_{tetR}
Canine	Other / mixed	*Escherichia coli*	1	0	AMP AUG KF CPD POTS	0	bla_{DHA}	SAMN16279747	5879	648	$bla_{\text{OXA-1}}$ Flq_{qnrB4} Sul_{sul1} Sul_{sul2} Tmt_{tetR}
Canine	Anal region	*Escherichia coli*	1	1	AMP AUG KF CPD	0	bla_{CIT} *aac(6')-Ib*	SAMN16279765	5879	648	$bla_{\text{CMY-111}}$ Flq_{qnrB4} Sul_{sul1} Tmt_{tetR}
Canine	Urine	*Escherichia coli*	1	0	AMP AUG KF CPD C ENF TET POTS	0	bla_{OXA} *aac(6')-Ib*	SAMN16278429	6870	648	$AGly_{\text{aadA5}}$ Phe_{catA1} Phe_{catB3} Sul_{sul1} Tet_{tetB} Tmt_{tetR}
Canine	Other / mixed	*Escherichia coli*	1	0	AMP KF CPD C POTS	0	bla_{OXA} bla_{CIT}	SAMN16279748	10064	Unknown	$AGly_{\text{aadA1-pm}}$ $bla_{\text{CMY-111}}$ $bla_{\text{SHV-12}}$ $bla_{\text{TEM-105}}$ Phe_{floR} Sul_{sul1} Sul_{sul2}
Canine	Other / mixed	*Escherichia coli*	1	0	AMP AUG KF CPD ENF TET POTS	0	bla_{CIT} *qnrB* *aac(6')-Ib*	SAMN16278390	Unknown	Unknown	Unknown
Canine	Urine	*Escherichia coli*	1	0	AMP AUG KF CPD	0		SAMN16278404	Unknown	Unknown	Unknown
Canine	Urine	*Escherichia coli*	1	0	AMP AUG KF CPD	0		SAMN16278417	Unknown	Unknown	Unknown
Canine	Other / mixed	*Escherichia coli*	1	1	AMP AUG KF CPD TET	0	bla_{TEM} bla_{CIT} *aac(6')-Ib*	SAMN16279769	Unknown	Unknown	Unknown
Canine	Other / mixed	*Escherichia coli*	0	0	ENF	0		Not tested	Not tested	Not tested	Not tested

Animal species	Sample type/site	Referring laboratory reported bacterial species	Confirmed E. coli	ESBL phenotype	Resistance phenotype	PCR ST131:O25b	PCR plasmid-mediated AMR genes	GenBank accession number	WGS Sequence type	WGS Clonal complex	WGS plasmid-mediated AMR genes
Canine	Oronasal / respiratory	*Klebsiella pneumoniae*	0	0	AMP AUG KF CPD C ENF	Not tested	bla_{SHV} bla_{DHA}	Not tested	Not tested	Not tested	Not tested
Canine	Ear	*Proteus* species	0	1	AMP AUG KF CPD ENF GM TET POTS	Not tested	bla_{TEM} bla_{CIT}	Not tested	Not tested	Not tested	Not tested
Canine	Other / mixed	*Klebsiella pneumoniae*	0	1	AMP KF CPD GM TET POTS	Not tested	bla_{SHV} bla_{OXA}	Not tested	Not tested	Not tested	Not tested
Canine	Other / mixed	*Enterobacter cloacae* complex	0	0	AUG KF	Not tested		Not tested	Not tested	Not tested	Not tested
Canine	Urine	*Escherichia coli*	0	0	AMP AUG KF	0		Not tested	Not tested	Not tested	Not tested
Canine	Urine	*Proteus mirabilis*	0	0	AMP C ENF TET POTS	Not tested	bla_{TEM}	Not tested	Not tested	Not tested	Not tested
Canine	Urine	*Escherichia coli*	0	0	AMP	0	bla_{SHV}	Not tested	Not tested	Not tested	Not tested
Canine	Other / mixed	*Morganella morganii*	0	0	AMP AUG KF TET	Not tested		Not tested	Not tested	Not tested	Not tested
Canine	Ear	*Citrobacter koseri*	0	1	AMP AUG KF CPD ENF TET POTS	Not tested	bla_{TEM} bla_{SHV} bla_{OXA} $bla_{\text{CTX-M-15}}$ bla_{CIT} *aac(6')-Ib*	Not tested	Not tested	Not tested	Not tested
Canine	Urine	*Klebsiella pneumoniae*	0	1	AMP AUG KF CPD ENF TET POTS	Not tested	bla_{TEM} bla_{SHV} bla_{OXA} $bla_{\text{CTX-M-15}}$ bla_{CIT} *aac(6')-Ib*	Not tested	Not tested	Not tested	Not tested
Canine	Urine	*Proteus mirabilis*	0	0	GM TET	Not tested	bla_{TEM}	Not tested	Not tested	Not tested	Not tested
Canine	Urine	*Proteus mirabilis*	0	0	C TET	Not tested		Not tested	Not tested	Not tested	Not tested
Canine	Urine	Enterobacter_asburiae	0	0	AMP AUG KF CPD POTS	Not tested	bla_{TEM} bla_{ACC}	Not tested	Not tested	Not tested	Not tested
Canine	Urine	*Enterobacter cloacae* complex	0	0		Not tested		Not tested	Not tested	Not tested	Not tested
Canine	Urine	*Escherichia coli*	0	0	AMP AUG KF POTS	0	bla_{TEM}	Not tested	Not tested	Not tested	Not tested
Canine	Ear	*Enterobacter cloacae* complex	0	0	C	Not tested		Not tested	Not tested	Not tested	Not tested
Feline	Urine	*Serratia marcescens*	0	0	AMP AUG KF TET	Not tested		Not tested	Not tested	Not tested	Not tested
Canine	Oronasal / respiratory	*Enterobacter cloacae* complex	1	0		Not tested		Not tested	Not tested	Not tested	Not tested
Canine	Ear	*Proteus mirabilis*	0	0	C TET POTS	Not tested	bla_{TEM}	Not tested	Not tested	Not tested	Not tested
Canine	Urine	*Escherichia coli*	1	0		0		Not tested	Not tested	Not tested	Not tested
Canine	Urine	*Enterobacter cloacae* complex	0	0		Not tested		Not tested	Not tested	Not tested	Not tested
Canine	Urine	*Escherichia coli*	1	0		0		Not tested	Not tested	Not tested	Not tested
Feline	Other / mixed	*Klebsiella pneumoniae*	1	1	AMP KF CPD ENF TET POTS	Not tested	$bla_{\text{CTX-M-15}}$ *aac(6')-Ib*	Not tested	Not tested	Not tested	Not tested
Canine	Ear	*Klebsiella pneumoniae*	0	1	AMP KF CPD C ENF GM TET POTS	Not tested	bla_{TEM} bla_{OXA} $bla_{\text{CTX-M-15}}$ *qnrB* *aac(6')-Ib*	Not tested	Not tested	Not tested	Not tested
Feline	Other / mixed	*Enterobacter cloacae* complex	0	0	AMP AUG KF CPD	Not tested		Not tested	Not tested	Not tested	Not tested

Animal species	Sample type/site	Referring laboratory reported bacterial species	Confirmed E. coli	ESBL phenotype	Resistance phenotype	PCR ST131:O25b	PCR plasmid-mediated AMR genes	GenBank accession number	WGS Sequence type	WGS Clonal complex	WGS plasmid-mediated AMR genes
Canine	Urine	*Proteus mirabilis*	0	0	AMP C TET POTS	Not tested	bla_{TEM}	Not tested	Not tested	Not tested	Not tested
Feline	Urine	*Serratia marcescens*	0	0	AMP AUG KF TET	Not tested		Not tested	Not tested	Not tested	Not tested
Canine	Urine	*Proteus mirabilis*	0	0	AMP C ENF GM TET POTS	Not tested	bla_{TEM}	Not tested	Not tested	Not tested	Not tested
Canine	Urine	*Proteus mirabilis*	0	0	ENF TET	Not tested		Not tested	Not tested	Not tested	Not tested
Canine	Urine	*Enterobacter cloacae* complex	0	0	AMP AUG KF	Not tested	bla_{ACC}	Not tested	Not tested	Not tested	Not tested
Canine	Other / mixed	*Klebsiella* species	1	0	AMP AUG C ENF TET POTS	Not tested	bla_{TEM} *aac(6')-Ib*	Not tested	Not tested	Not tested	Not tested
Canine	Other / mixed	*Proteus mirabilis*	0	1	AMP AUG KF CPD ENF GM TET POTS	Not tested	bla_{TEM} bla_{CIT}	Not tested	Not tested	Not tested	Not tested
Canine	Urine	*Proteus mirabilis*	0	0	TET	Not tested		Not tested	Not tested	Not tested	Not tested
Canine	Other / mixed	Serratia_marcescens	0	0	KF TET	Not tested		Not tested	Not tested	Not tested	Not tested
Canine	Urine	*Proteus mirabilis*	0	1	AMP KF CPD C ENF TET	Not tested	bla_{CTX-M}	Not tested	Not tested	Not tested	Not tested
Canine	Urine	*Klebsiella pneumoniae*	0	0	AMP AUG KF CPD ENF	Not tested	bla_{SHV} bla_{OXA} bla_{DHA} *aac(6')-Ib*	Not tested	Not tested	Not tested	Not tested
Canine	faeces	Salmonella_species	0	0	AMP TET	Not tested	bla_{TEM}	Not tested	Not tested	Not tested	Not tested
Feline	Other / mixed	*Enterobacter cloacae* complex	0	0	AUG KF TET	Not tested		Not tested	Not tested	Not tested	Not tested
Canine	Urine	*Enterobacter cloacae* complex	0	0	AMP AUG KF C	Not tested		Not tested	Not tested	Not tested	Not tested
Canine	Oronasal / respiratory	*Escherichia coli*	0	0	AMP C ENF GM TET POTS	0	bla_{SHV}	Not tested	Not tested	Not tested	Not tested
Canine	Other / mixed	*Klebsiella pneumoniae*	0	0	AMP AUG KF CPD C ENF GM TET POTS	Not tested		Not tested	Not tested	Not tested	Not tested
Canine	Ear	*Klebsiella pneumoniae*	0	1	AMP KF CPD C ENF GM TET POTS	Not tested	bla_{TEM} bla_{SHV} bla_{OXA} $bla_{CTX-M-15}$ *qnrB aac(6')-Ib*	Not tested	Not tested	Not tested	Not tested
Canine	Other / mixed	*Klebsiella pneumoniae*	0	0	AMP KF CPD ENF	Not tested	bla_{SHV} bla_{OXA} bla_{DHA} *aac(6')-Ib*	Not tested	Not tested	Not tested	Not tested
Canine	Urine	*Escherichia coli*	0	1	AMP AUG KF CPD C GM TET POTS	0	bla_{TEM}	Not tested	Not tested	Not tested	Not tested
Canine	Urine	*Proteus mirabilis*	0	0	AMP TET POTS	Not tested	bla_{TEM}	Not tested	Not tested	Not tested	Not tested
Canine	Urine	*Proteus mirabilis*	0	0	TET	Not tested		Not tested	Not tested	Not tested	Not tested
Canine	Urine	*Klebsiella pneumoniae*	0	0	AMP	Not tested		Not tested	Not tested	Not tested	Not tested
Canine	Urine	*Escherichia coli*	0	0	AMP	0		Not tested	Not tested	Not tested	Not tested
Canine	Urine	*Escherichia coli*	0	1	AMP KF CPD ENF	0	bla_{SHV} bla_{DHA} *aac(6')-Ib*	Not tested	Not tested	Not tested	Not tested
Canine	Urine	*Escherichia coli*	0	0	GM TET	0		Not tested	Not tested	Not tested	Not tested

Key: AMP = ampicillin; C = Chloramphenicol; AUG = clavulanic acid potentiated amoxicillin; KF = Cephalothin; CPD = Cefpodoxime; ENF = Enrofloxacin; GM = Gentamicin; TET = Tetracycline; POTS = Trimethoprim potentiated sulphonamide

Supplementary material 5: Univariable results from a mixed effects logistic regression model, exploring odds of multi-drug resistant (MDR) *E. coli* at an isolate-level against a range of putative risk factors for dogs. Veterinary practice site was included as a random effect in all models.

Variable	Category	β	SE [a]	OR [b] (CI) [c]	*P*
Sex	Female (Intercept)	-1.94	0.24	1.00	
	Male	-0.03	0.04	0.97 (1.89-1.06)	0.51
	Unspecified	0.26	0.12	1.29 (1.22-1.64)	0.03
Neuter status	Un-neutered (Intercept)	-1.99	0.25	1.00	
	Neutered	0.02	0.06	1.02 (0.92-1.14)	0.72
	Unspecified	0.10	0.07	1.10 (4.96-1.27)	0.18
Genetic breed group [d]	Retriever (Intercept)	-2.03	0.25	1.00	
	Ancient / spitz	0.15	0.16	1.15 (8.84-1.60)	0.37
	Crossbreed	-0.01	0.08	0.99 (3.85-1.16)	0.93
	Herding	-0.17	0.12	0.84 (0.67-1.05)	0.13
	Mastiff-like	0.15	0.09	1.16 (6.98-1.38)	0.08
	Scent hound	0.20	0.13	1.21 (8.95-1.57)	0.13
	Sight hound	-0.06	0.19	0.93 (9.65-1.35)	0.74
	Small terrier	0.06	0.10	1.05 (8.87-1.28)	0.56
	Spaniel	0.07	0.08	1.07 (6.92-1.26)	0.36
	Toy	0.24	0.12	1.26 (5.99-1.61)	0.06
	Not yet genetically classified	0.13	0.09	1.13 (9.96-1.35)	0.13
	Unknown breed	0.14	0.09	1.15 (0.96-1.37)	0.12
	Working dog	0.38	0.11	1.46 (1.28-1.80)	<0.01
Urban / rural status	Urban (Intercept)	-1.94	0.24	1.00	
	Rural	-0.02	0.07	0.98 (4.87-1.12)	0.80
Species treated	Dog & cat (Intercept)	-1.95	0.24	1.00	
	Dog, cat & equine	0.26	0.16	1.29 (1.95-1.75)	0.10
	Dog, cat, equine & farm	-0.14	0.08	0.87 (4.75-1.02)	0.09
	Dog, cat & farm	0.20	0.15	1.22 (5.92-1.63)	0.17
RCVS accreditation	Not accredited (Intercept)	-1.98	0.24	1.00	
	Accredited	0.07	0.05	1.07 (5.97-1.19)	0.16
RCVS hospital status	Not hospital (Intercept)	-1.98	0.24	1.00	
	Hospital	0.13	0.08	1.13 (6.98-1.32)	0.09
Emergency / out of hours provider	Not emergency / OOH (Intercept)	-1.95	0.24	1.00	
	Emergency / OOH provider	0.04	0.12	1.04 (3.82-1.33)	0.74
Referrals only	Not referrals-only site (Intercept)	-2.01	0.25	0.13 (4.08-0.22)	<0.01
	Referrals-only site	0.98	0.19	2.66 (1.83-3.86)	<0.01
	Mixed site	-0.28	0.26	0.75 (2.45-1.26)	0.28
RCVS AVP [e]	No RCVS AVPs on site (Intercept)	-1.95	0.24	1.00	
	RCVS AVPs on site	0.00	0.06	1.00 (3.89-1.14)	0.97
RCVS Specialist	No RCVS specialist on site (Intercept)	-2.03	0.25	1.00	
	RCVS specialist on site	0.48	0.11	1.62 (1.32-2.30)	<0.01
Veterinary nurse training	Not VN training site (Intercept)	-1.96	0.24	1.00	
	VN training site	0.01	0.07	1.01 (3.89-1.16)	0.85
Sites per postcode	Single site (Intercept)	-1.94	0.24	1.00	
	Multiple sites	-0.20	0.15	0.81 (5.61-1.09)	0.16
Sampling type / site	Urine (Intercept)	-2.12	0.23	1.00	
	Anal region (including anal sacs)	0.32	0.06	1.37 (1.21-1.56)	<0.01
	Ear(s)	-1.15	0.14	0.31 (7.24-0.42)	<0.01
	Faeces	-0.02	0.20	0.98 (4.66-1.46)	0.94
	Oronasopharyngeal & respiratory	0.58	0.14	1.79 (1.37-2.33)	<0.01
	Other sites or mixed	0.64	0.05	1.89 (1.74-2.11)	<0.01

[a] Standard error
[b] Odds ratio
[c] 95% Confidence interval
[d] Vonholdt et al. (2010)
[e] Royal College of Veterinary Surgeons Advanced Veterinary Practitioner

Supplementary material 6: Univariable results from a mixed effects logistic regression model, exploring odds of multi-drug resistant (MDR) *E. coli* at an isolate-level against a range of putative risk factors for cats. Veterinary practice site was included as a random effect in all models.

Variable	Category	β	SE [a]	OR [b] (CI) [c]	*P*
Sex	Female (Intercept)	-2.71	0.28	1.00	
	Male	0.20	0.08	1.21 (1.54-1.41)	0.01
	Unspecified	0.22	0.20	1.25 (2.84-1.87)	0.27
Neuter status	Un-neutered (Intercept)	-2.58	0.31	1.00	
	Neutered	-0.07	0.14	0.93 (6.72-1.22)	0.62
	Unspecified	-0.04	0.17	0.95 (7.68-1.34)	0.80
Genetic breed group [d]	West Europe (Intercept)	-2.51	0.31	1.00	
	Asian	0.03	0.20	1.03 (1.69-1.53)	0.88
	Crossbreed	-0.13	0.15	0.88 (1.66-1.18)	0.40
	Not yet genetically classified	-0.39	0.25	0.67 (8.42-1.10)	0.12
	Unknown breed	-0.25	0.18	0.77 (8.55-1.11)	0.16
Urban / rural status	Urban (Intercept)	-2.59	0.27	1.00	
	Rural	-0.16	0.13	0.85 (5.66-1.11)	0.24
Species treated	Dog & cat (Intercept)	-2.62	0.28	1.00	
	Dog, cat & equine	0.02	0.29	1.01 (7.58-1.80)	0.95
	Dog, cat, equine & farm	-0.04	0.16	0.96 (1.70-1.32)	0.80
	Dog, cat & farm	-0.37	0.31	0.69 (4.38-1.27)	0.23
RCVS accreditation	Not accredited (Intercept)	-2.74	0.29	1.00	
	Accredited	0.20	0.09	1.21 (1.72-1.46)	0.03
RCVS hospital status	Not hospital (Intercept)	-2.65	0.28	1.00	
	Hospital	0.04	0.13	1.04 (0.80-1.35)	0.77
Emergency / out of hours provider	Not emergency / OOH (Intercept)	-2.65	0.28	1.00	
	Emergency / OOH provider	0.17	0.21	1.18 (5.78-1.80)	0.42
Referrals only	Not referrals-only site (Intercept)	-2.68	0.29	1.00	
	Referrals-only site	0.51	0.36	1.66 (8.82-3.38)	0.16
	Mixed site	0.36	0.40	1.43 (7.66-3.13)	0.36
RCVS AVP [e]	No RCVS AVPs on site (Intercept)	-2.64	0.28	1.00	
	RCVS AVPs on site	0.02	0.11	1.01 (6.82-1.26)	0.89
RCVS Specialist	No RCVS specialist on site (Intercept)	-2.72	0.30	1.00	
	RCVS specialist on site	0.34	0.19	1.41 (1.97-2.06)	0.07
Veterinary nurse training	Not VN training site (Intercept)	-2.58	0.29	1.00	
	VN training site	-0.07	0.12	0.93 (4.74-1.18)	0.56
Sites per postcode	Single site (Intercept)	-2.64	0.28	1.00	
	Multiple sites	0.29	0.23	1.34 (2.86-2.10)	0.20
Sampling type / site	Urine (Intercept)	-2.74	0.30	1.00	
	Anal region (including anal sacs)	0.10	0.30	1.10 (7.62-1.98)	0.73
	Ear(s)	-1.99	0.68	0.13 (7.04-0.52)	0.00
	Faeces	0.12	0.27	1.12 (3.66-1.92)	0.67
	Oronasopharyngeal & respiratory	0.53	0.20	1.69 (1.15-2.49)	0.01
	Other sites or mixed	0.57	0.12	1.77 (1.39-2.24)	<0.01

[a] Standard error
[b] Odds ratio
[c] 95% Confidence interval
[d] Lipinski et al. (2008)
[e] Royal College of Veterinary Surgeons Advanced Veterinary Practitioner

Supplementary material 7: Univariable results from a mixed effects logistic regression model, exploring odds of fluoroquinolone resistance in *E. coli* at an isolate-level against a range of putative risk factors for dogs. Veterinary practice site was included as a random effect in all models.

Variable	Category	β	SE [a]	OR [b] (CI) [c]	*P*
Sex	Female (Intercept)	-3.34	0.07	0.04 (0.03-0.04)	
	Male	0.05	0.07	1.06 (0.92-1.21)	0.45
	Unspecified	0.35	0.19	1.42 (0.98-2.06)	0.07
Neuter status	Un-neutered (Intercept)	-3.43	0.10	0.03 (0.03-0.04)	
	Neutered	0.19	0.09	1.21 (1.02-1.46)	0.04
	Unspecified	0.03	0.12	1.03 (0.81-1.30)	0.84
Genetic breed group [d]	Retriever (Intercept)	-3.21	0.10	0.04 (0.03-0.05)	
	Ancient / spitz	-0.01	0.26	0.99 (0.60-1.64)	0.97
	Crossbreed	-0.32	0.13	0.72 (0.56-0.93)	0.01
	Herding	-0.23	0.18	0.79 (0.56-1.13)	0.20
	Mastiff-like	-0.15	0.14	0.86 (0.66-1.13)	0.28
	Scent hound	0.19	0.19	1.21 (0.84-1.75)	0.30
	Sight hound	-0.51	0.33	0.60 (0.32-1.14)	0.12
	Small terrier	-0.35	0.17	0.70 (0.51-0.970)	0.03
	Spaniel	-0.10	0.12	0.91 (0.71-1.15)	0.41
	Toy	0.05	0.20	1.05 (0.72-1.54)	0.79
	Not yet genetically classified	0.06	0.13	1.06 (0.83-1.37)	0.64
	Unknown breed	-0.03	0.14	0.97 (0.74-1.29)	0.85
	Working dog	0.03	0.16	1.03 (0.75-1.43)	0.84
Urban / rural status	Urban (Intercept)	-3.31	0.07	0.04 (0.03-0.04)	
	Rural	0.02	0.12	1.02 (0.81-1.28)	0.86
Species treated	Dog & cat (Intercept)	-3.28	0.07	0.04 (0.03-0.04)	
	Dog, cat & equine	0.26	0.29	1.29 (0.74-2.26)	0.37
	Dog, cat, equine & farm	-0.20	0.15	0.82 (0.62-1.09)	0.17
	Dog, cat & farm	-0.21	0.29	0.81 (0.46-1.42)	0.47
RCVS accreditation	Not accredited (Intercept)	-3.38	0.08	0.03 (0.03-0.04)	
	Accredited	0.14	0.10	1.15 (0.95-1.38)	0.15
RCVS hospital status	Not hospital (Intercept)	-3.36	0.07	0.04 (0.03-0.04)	
	Hospital	0.37	0.14	1.44 (1.10-1.90)	0.01
Emergency / out of hours provider	Not emergency / OOH (Intercept)	-3.30	0.07	0.04 (0.03-0.04)	
	Emergency / OOH provider	0.00	0.24	1.00 (0.63-1.59)	0.99
Referrals only	Not referrals-only site (Intercept)	-3.32	0.07	0.04 (0.03-0.04)	
	Referrals-only site	1.75	0.33	5.74 (3.03-10.88)	<0.01
	Mixed site	-0.10	0.49	0.91 (0.35-2.37)	0.84
RCVS AVP [e]	No RCVS AVPs on site (Intercept)	-3.31	0.07	0.04 (0.03-0.04)	
	RCVS AVPs on site	0.01	0.12	1.01 (0.80-1.27)	0.94
RCVS Specialist	No RCVS specialist on site (Intercept)	-3.35	0.07	0.04 (0.03-0.04)	
	RCVS specialist on site	1.05	0.18	2.85 (2.01-4.04)	<0.01
Veterinary nurse training	Not VN training site (Intercept)	-3.42	0.12	0.03 (0.03-0.04)	
	VN training site	0.14	0.12	1.15 (0.90-1.46)	0.27
Sites per postcode	Single site (Intercept)	-3.30	0.07	0.04 (0.03-0.04)	
	Multiple sites	-0.08	0.27	0.92 (0.54-1.57)	0.76
Sampling type / site	Urine (Intercept)	-3.50	0.07	0.03 (0.03-0.04)	
	Anal region (including anal sacs)	0.36	0.11	1.43 (1.16-1.75)	<0.01
	Ear(s)	0.51	0.13	1.67 (1.30-2.14)	<0.01
	Faeces	-0.62	0.43	0.54 (0.23-1.25)	0.15
	Oronasopharyngeal & respiratory	0.51	0.21	1.67 (1.10-2.53)	0.02
	Other sites or mixed	0.57	0.09	1.76 (1.49-2.09)	<0.01

[a] Standard error
[b] Odds ratio
[c] 95% Confidence interval
[d] Vonholdt et al. (2010)
[e] Royal College of Veterinary Surgeons Advanced Veterinary Practitioner

Supplementary material 8: Univariable results from a mixed effects logistic regression model, exploring odds of fluoroquinolone resistance in *E. coli* at an isolate-level against a range of putative risk factors for cats. Veterinary practice site was included as a random effect in all models.

Variable	Category	β	SE [a]	OR [b] (CI) [c]	*P*
Sex	Female (Intercept)	-8.42	0.52	0.00 (0.00-0.00)	
	Male	0.48	0.22	1.61 (1.05-2.48)	0.03
	Unspecified	0.65	0.56	1.91 (0.63-5.77)	0.25
Neuter status	Un-neutered (Intercept)	-8.20	0.65	0.00 (0.00-0.00)	
	Neutered	0.04	0.44	1.04 (0.43-2.47)	0.94
	Unspecified	-0.24	0.55	0.79 (0.27-2.32)	0.66
Genetic breed group [d]	West Europe (Intercept)	-7.52	0.57	0.00 (0.00-0.00)	
	Asian	-1.05	0.55	0.35 (0.12-1.03)	0.06
	Crossbreed	-0.85	0.36	0.43 (0.21-0.87)	0.02
	Not yet genetically classified	-2.04	0.83	0.13 (0.03-0.66)	0.01
	Unknown breed	-0.80	0.48	0.45 (0.18-1.15)	0.10
Urban / rural status	Urban (Intercept)	-8.23	0.52	0.00 (0.00-0.00)	
	Rural	0.22	0.58	1.24 (0.40-3.87)	0.718
Species treated	Dog & cat (Intercept)	-8.20	0.52	0.00 (0.00-0.00)	
	Dog, cat & equine	-0.11	1.44	0.89 (0.05-14.99)	0.94
	Dog, cat, equine & farm	0.08	0.74	1.08 (0.26-4.60)	0.91
	Dog, cat & farm	-0.29	1.51	0.75 (0.04-14.24)	0.85
RCVS accreditation	Not accredited (Intercept)	-8.19	0.56	0.00 (0.00-0.00)	
	Accredited	-0.02	0.44	0.98 (0.41-2.33)	0.96
RCVS hospital status	Not hospital (Intercept)	-8.24	0.52	0.00 (0.00-0.00)	
	Hospital	0.37	0.60	1.44 (0.44-4.69)	0.54
Emergency / out of hours provider	Not emergency / OOH (Intercept)	-8.18	0.51	0.00 (0.00-0.00)	
	Emergency / OOH provider	-0.40	1.19	0.67 (0.07-6.99)	0.74
Referrals only	Not referrals-only site (Intercept)	-8.20	0.00	0.00 (0.00-0.00)	
	Referrals-only site	1.27	1.41	3.56 (0.22-56.51)	0.37
	Mixed site	-0.58	2.65	0.56 (0.00-100.38)	0.83
RCVS AVP [e]	No RCVS AVPs on site (Intercept)	-8.22	0.52	0.00 (0.00-0.00)	
	RCVS AVPs on site	0.09	0.53	1.09 (0.38-3.10)	0.87
RCVS Specialist	No RCVS specialist on site (Intercept)	-8.17	0.52	0.00 (0.00-0.00)	
	RCVS specialist on site	1.35	0.75	3.87 (0.88-16.97)	0.07
Veterinary nurse training	Not VN training site (Intercept)	-8.21	0.70	0.00 (0.00-0.00)	
	VN training site	0.01	0.59	1.01 (0.32-3.18)	0.99
Sites per postcode	Single site (Intercept)	-8.19	0.51	0.00 (0.00-0.00)	
	Multiple sites	-0.38	1.33	0.68 (0.05-9.25)	0.77
Sampling type / site	Urine (Intercept)	-8.23	0.53	0.00 (0.00-0.00)	
	Anal region (including anal sacs)	1.49	0.66	4.45 (1.23-16.10)	0.02
	Ear	1.01	0.71	2.75 (0.69-11.02)	0.15
	Faeces	0.62	0.86	1.87 (0.35-10.04)	0.47
	Oronasopharyngeal & respiratory	0.52	0.58	1.68 (0.54-5.22)	0.37
	Other sites or mixed	0.88	0.32	2.40 (1.28-4.52)	0.01

[a] Standard error
[b] Odds ratio
[c] 95% Confidence interval
[d] Lipinski et al. (2008)
[e] Royal College of Veterinary Surgeons Advanced Veterinary Practitioner

Supplementary material 9: Univariable results from a mixed effects logistic regression model, exploring odds of potentiated penicillin resistance in *E. coli* at an isolate-level against a range of putative risk factors for dogs. Veterinary practice site and laboratory site were included as random effects in all models.

Variable	Category	β	SE [a]	OR [b] (CI) [c]	*P*
Sex	Female (Intercept)	-1.47	0.23	0.23 (0.15-0.36)	
	Male	-0.02	0.05	0.99 (0.90-1.08)	0.74
	Unspecified	0.28	0.13	1.32 (1.04-1.69)	0.03
Neuter status	Un-neutered (Intercept)	-1.49	0.23	0.23 (0.14-0.35)	
	Neutered	0.01	0.06	1.01 (0.90-1.14)	0.83
	Unspecified	0.04	0.08	1.04 (0.90-1.21)	0.59
Genetic breed group [d]	Retriever (Intercept)	-1.61	0.23	0.20 (0.13-0.31)	
	Ancient / spitz	0.14	0.17	1.15 (0.81-1.61)	0.44
	Crossbreed	0.10	0.09	1.10 (0.93-1.30)	0.26
	Herding	-0.20	0.13	0.82 (0.64-1.05)	0.11
	Mastiff-like	0.35	0.09	1.42 (1.20-1.70)	<0.01
	Scent hound	0.24	0.14	1.27 (0.98-1.66)	0.07
	Sight hound	0.04	0.19	1.04 (0.71-1.51)	0.85
	Small terrier	0.26	0.10	1.30 (1.07-1.58)	0.01
	Spaniel	0.11	0.08	1.11 (0.94-1.31)	0.21
	Toy	0.38	0.13	1.47 (1.14-1.89)	<0.01
	Not yet genetically classified	0.15	0.09	1.16 (0.97-1.38)	0.11
	Unknown breed	0.12	0.10	1.13 (0.93-1.37)	0.21
	Working dog	0.33	0.11	1.39 (1.11-1.72)	<0.01
Urban / rural status	Urban (Intercept)	-1.45	0.23	0.24 (0.15-0.37)	
	Rural	-0.08	0.07	0.93 (0.81-1.05)	0.23
Species treated	Dog & cat (Intercept)	-1.45	0.22	0.24 (0.15-0.36)	
	Dog, cat & equine	0.10	0.16	1.11 (0.81-1.50)	0.52
	Dog, cat, equine & farm	-0.21	0.08	0.81 (0.69-0.95)	0.01
	Dog, cat & farm	0.08	0.14	1.08 (0.82-1.43)	0.57
RCVS accreditation	Not accredited (Intercept)	-1.49	0.23	0.23 (0.15-0.35)	
	Accredited	0.03	0.05	1.03 (0.93-1.14)	0.53
RCVS hospital status	Not hospital (Intercept)	-1.50	0.22	0.22 (0.15-0.35)	
	Hospital	0.10	0.07	1.10 (0.96-1.27)	0.18
Emergency / out of hours provider	Not emergency / OOH (Intercept)	-1.47	0.22	0.23 (0.15-0.36)	
	Emergency / OOH provider	0.05	0.12	1.06 (0.84-1.33)	0.65
Referrals only	Not referrals-only site (Intercept)	-1.53	0.20	0.22 (0.15-0.32)	
	Referrals-only site	0.95	0.17	2.58 (1.86-3.57)	<0.01
	Mixed site	-0.35	0.28	0.71 (0.41-1.22)	0.21
RCVS AVP [e]	No RCVS AVPs on site (Intercept)	-1.50	0.22	0.22 (0.15-0.35)	
	RCVS AVPs on site	0.09	0.06	1.09 (0.97-1.23)	0.16
RCVS Specialist	No RCVS specialist on site (Intercept)	-1.57	0.20	0.21 (0.14-0.31)	<0.01
	RCVS specialist on site	0.52	0.09	1.69 (1.40-2.03)	<0.01
Veterinary nurse training	Not VN training site (Intercept)	-1.46	0.23	0.23 (0.15-0.37)	
	VN training site	-0.01	0.07	0.99 (0.87-1.14)	0.93
Sites per postcode	Single site (Intercept)	-1.46	0.22	0.23 (0.15-0.36)	
	Multiple sites	-0.18	0.14	0.84 (0.63-1.11)	0.22
Sampling type / site	Urine (Intercept)	-1.71	0.20	0.18 (0.12-0.27)	
	Anal region (including anal sacs)	0.19	0.07	1.21 (1.05-1.38)	0.01
	Ear(s)	-0.02	0.09	0.98 (0.82-1.18)	0.86
	Faeces	0.08	0.22	1.08 (0.71-1.66)	0.71
	Oronasopharyngeal & respiratory	0.43	0.14	1.54 (1.16-2.03)	<0.01
	Other sites or mixed	0.67	0.06	1.95 (1.75-2.18)	<0.01

[a] Standard error
[b] Odds ratio
[c] 95% Confidence interval
[d] Vonholdt et al. (2010)
[e] Royal College of Veterinary Surgeons Advanced Veterinary Practitioner

Supplementary material 10: Univariable results from a mixed effects logistic regression model, exploring odds of potentiated penicillin resistance in *E. coli* at an isolate-level against a range of putative risk factors for cats. Veterinary practice site and laboratory site were included as random effects in all models.

Variable	Category	β	SE [a]	OR [b] (CI) [c]	*P*
Sex	Female (Intercept)	-1.76	0.48	0.17 (0.07-0.44)	
	Male	0.17	0.08	1.18 (1.02-1.38)	0.03
	Unspecified	0.17	0.20	1.19 (0.80-1.77)	0.40
Neuter status	Un-neutered (Intercept)	-1.68	0.50	0.19 (0.07-0.49)	
	Neutered	-0.01	0.14	0.99 (0.75-1.31)	0.96
	Unspecified	-0.03	0.18	0.97 (0.69-1.37)	0.86
Genetic breed group [d]	West Europe (Intercept)	-1.54	0.49	0.22 (0.08-0.56)	
	Asian	-0.07	0.20	0.93 (0.63-1.38)	0.72
	Crossbreed	-0.16	0.15	0.85 (0.64-1.14)	0.28
	Not yet genetically classified	-0.30	0.24	0.74 (0.46-1.19)	0.21
	Unknown breed	-0.33	0.18	0.72 (0.50-1.03)	0.07
Urban / rural status	Urban (Intercept)	-1.67	0.48	0.19 (0.07-0.48)	
	Rural	-0.08	0.12	0.92 (0.72-1.17)	0.50
Species treated	Dog & cat (Intercept)	-1.67	0.47	0.19 (0.08-0.47)	
	Dog, cat & equine	-0.21	0.30	0.81 (0.46-1.45)	0.48
	Dog, cat, equine & farm	-0.02	0.15	0.98 (0.73-1.33)	0.91
	Dog, cat & farm	-0.38	0.29	0.69 (0.39-1.21)	0.20
RCVS accreditation	Not accredited (Intercept)	-1.76	0.47	0.17 (0.07-0.43)	
	Accredited	0.13	0.09	1.13 (0.95-1.35)	0.16
RCVS hospital status	Not hospital (Intercept)	-1.68	0.48	0.19 (0.07-0.47)	
	Hospital	-0.03	0.12	0.97 (0.76-1.23)	0.78
Emergency / out of hours provider	Not emergency / OOH (Intercept)	-1.72	0.47	0.18 (0.07-0.45)	
	Emergency / OOH provider	0.19	0.20	1.21 (0.82-1.78)	0.34
Referrals only	Not referrals-only site (Intercept)	-1.72	0.46	0.18 (0.07-0.44)	<0.01
	Referrals-only site	0.32	0.33	1.38 (0.73-2.62)	0.32
	Mixed site	0.41	0.38	1.51 (0.71-3.18)	0.28
RCVS AVP [e]	No RCVS AVPs on site (Intercept)	-1.69	0.48	0.19 (0.07-0.47)	
	RCVS AVPs on site	-0.01	0.11	0.99 (0.81-1.22)	0.93
RCVS Specialist	No RCVS specialist on site (Intercept)	-1.73	0.46	0.18 (0.07-0.44)	
	RCVS specialist on site	0.17	0.18	1.19 (0.84-1.68)	0.33
Veterinary nurse training	Not VN training site (Intercept)	-1.63	0.48	0.20 (0.08-0.51)	
	VN training site	-0.08	0.12	0.92 (0.74-1.16)	0.48
Sites per postcode	Single site (Intercept)	-1.70	0.48	0.18 (0.07-0.46)	
	Multiple sites	0.39	0.21	1.48 (0.98-2.25)	0.06
Sampling type / site	Urine (Intercept)	-1.82	0.43	0.16 (0.07-0.38)	
	Anal region (including anal sacs)	-0.12	0.32	0.89 (0.47-1.67)	0.72
	Ear	-0.40	0.34	0.67 (0.35-1.30)	0.24
	Faeces	-0.18	0.32	0.83 (0.45-1.54)	0.56
	Oronasopharyngeal & respiratory	0.40	0.20	1.49 (1.01-2.21)	0.05
	Other sites or mixed	0.52	0.12	1.67 (1.32-2.12)	<0.01

[a] Standard error
[b] Odds ratio
[c] 95% Confidence interval
[d] Lipinski et al. (2008)
[e] Royal College of Veterinary Surgeons Advanced Veterinary Practitioner

Supplementary material 11: Multi-drug resistance. Multivariable mixed effects logistic model results, displaying risk factors significantly associated with odds of an *E. coli* clinical isolate being classed as multi-drug resistant in dogs and cats. For both models, veterinary practice site and laboratory site were modelled as random effects. Significant categories within a variable are emboldened.

Random effect(s)		Fixed effects					
**Variable**	**Var [a] (SD) [b]**	**Variable**	**Category**	**β**	**SE [c]**	**OR [d] (CI) [e]**	***P***
			**Dogs**				
Practice	0.17 (0.41)	Intercept		-2.29	0.26		
Laboratory	0.23 (0.48)	Sex	Female	-	-	1.00	-
			Male	-0.08	0.05	0.92 (5.85-1.01)	0.09
			Unspecified	0.17	0.12	1.18 (6.93-1.51)	0.16
		Genetic breed group [f]	Retriever	-	-	1.00	-
			Ancient / spitz	0.22	0.17	1.24 (2.90-1.72)	0.19
			Crossbreed	-0.02	0.08	0.97 (6.83-1.15)	0.77
			Herding	-0.15	0.12	0.86 (5.69-1.09)	0.21
			Mastiff-like	0.15	0.09	1.16 (7.98-1.39)	0.08
			Scent hound	0.21	0.13	1.23 (0.95-1.59)	0.11
			Sight hound	-0.14	0.19	0.87 (0.60-1.25)	0.45
			Small terrier	0.13	0.10	1.13 (9.94-1.39)	0.19
			Spaniel	0.07	0.08	1.07 (3.92-1.26)	0.39
			**Toy**	**0.25**	**0.13**	**1.28 (1.00-1.64)**	**0.05**
			Not yet genetically classified	0.15	0.09	1.16 (5.98-1.38)	0.08
			Unknown breed	0.13	0.09	1.13 (9.95-1.36)	0.15
			**Working dog**	**0.36**	**0.11**	**1.44 (1.17-1.77)**	**<0.01**
		Referrals only	Not referrals-only site	-	-	1.00	-
			**Referrals-only site**	**0.61**	**0.20**	**1.83 (1.23-2.73)**	**<0.01**
			Mixed site	-0.35	0.26	0.70 (8.43-1.18)	0.19
		RCVS Specialist	No RCVS specialist on site	-	-	1.00	-
			**RCVS specialist on site**	**0.24**	**0.11**	**1.26 (1.51-1.58)**	**0.04**
		Species treated	Dog & cat	-	-	1.00	-
			Dog, cat & equine	0.25	0.15	1.28 (3.96-1.72)	0.10
			Dog, cat, equine & farm	-0.09	0.08	0.91 (6.79-1.07)	0.26
			Dog, cat & farm	0.25	0.14	1.27 (9.97-1.69)	0.08
		Sampling type / site	Urine	-	-	1.00	-
			**Anal region (including anal sacs)**	**0.32**	**0.07**	**1.37 (1.21-1.56)**	**<0.01**
			**Ear(s)**	**-1.16**	**0.14**	**0.31 (4.24-0.41)**	**<0.01**
			Faeces	-0.03	0.20	0.97 (5.66-1.45)	0.90
			**Oronasopharyngeal & respiratory**	**0.59**	**0.14**	**1.81 (1.39-2.37)**	**<0.01**
			**Other sites or mixed**	**0.62**	**0.06**	**1.85 (1.67-2.97)**	**<0.01**
			**Cats**				
Practice	0.48 (0.70)	Intercept		-2.98	0.32		
Laboratory	0.24 (0.49)	Sex	Female	-	-	1.00	-
			**Male**	**0.18**	**0.08**	**1.19 (1.53-1.39)**	**0.02**
			Unspecified	0.17	0.21	1.18 (1.79-1.76)	0.42
		RCVS accreditation	Not accredited	-	-	1.00	-
			**Accredited**	**0.20**	**0.09**	**1.22 (1.22-1.46)**	**0.03**
		RCVS Specialist	No RCVS specialist on site	-	-	1.00	-
			RCVS specialist on site	0.23	0.19	1.26 (4.87-1.84)	0.22
		Sampling type / site	Urine	-	-	1.00	-
			Anal region (including anal sacs)	0.09	0.30	1.09 (5.61-1.97)	0.76
			**Ear(s)**	**-2.01**	**0.68**	**0.13 (4.04-0.51)**	**<0.01**
			Faeces	0.09	0.28	1.09 (1.64-1.87)	0.75
			**Oronasopharyngeal & respiratory**	**0.49**	**0.20**	**1.62 (1.19-2.40)**	**0.01**
			**Other sites or mixed**	**0.55**	**0.12**	**1.74 (1.36-2.21)**	**<0.01**

[a] Variance
[b] Standard deviation
[c] Standard error
[d] Odds ratio
[e] 95% Confidence interval
[f] Vonholdt et al. (2010)
[g] Lipinski et al. (2008)

Supplementary material 12: Fluoroquinolone resistance. Multivariable mixed effects logistic models displaying risk factors significantly associated with odds of an *E. coli* clinical isolate being classed as fluoroquinolone resistant in dogs and cats. For both models, veterinary practice site was modelled as a random effect. Significant categories within a variable are emboldened.

Random effect(s)			Fixed effects					
**Variable**	**Var [a]**	**(SD) [b]**	**Variable**	**Category**	**β**	**SE [c]**	**OR [d] (CI) [e]**	***P***
				**Dogs**				
Practice	0.88	(0.94)	Intercept		-3.56	0.13	0.03 (0.02-0.04)	
			Sex	Female	-	-	1.00	-
				Male	-0.01	0.07	0.99 (0.86-1.14)	0.93
				**Unspecified**	**0.50**	**0.21**	**1.65 (1.09-2.50)**	**0.02**
			Neuter status	Un-neutered	-	-	1.00	-
				**Neutered**	**0.23**	**0.09**	**1.26 (1.05-1.52)**	**0.01**
				Unspecified	-0.12	0.13	0.89 (0.69-1.15)	0.36
			Genetic breed group [f]	Retriever	-	-	1.00	-
				Ancient / spitz	-0.01	0.26	0.99 (0.60-1.64)	0.97
				**Crossbreed**	**-0.38**	**0.13**	**0.68 (0.53-0.88)**	**<0.0**
				Herding	-0.19	0.18	0.83 (0.59-1.18)	0.30
				Mastiff-like	-0.19	0.14	0.83 (0.63-1.09)	0.18
				Scent hound	0.18	0.19	1.20 (0.83-1.74)	0.34
				Sight hound	-0.56	0.33	0.57 (0.30-1.08)	0.09
				**Small terrier**	**-0.42**	**0.17**	**0.66 (0.48-0.92)**	**0.01**
				Spaniel	-0.13	0.12	0.88 (0.69-1.12)	0.29
				Toy	-0.01	0.20	0.99 (0.67-1.45)	0.95
				Not yet genetically classified	0.03	0.13	1.03 (0.80-1.33)	0.81
				Unknown breed	-0.04	0.14	0.96 (0.72-1.27)	0.77
				Working dog	-0.03	0.17	0.97 (0.71-1.35)	0.87
			Referrals only	Not referrals-only site	-	-	1.00	-
				**Referrals-only site**	**0.99**	**0.36**	**2.69 (1.34-5.39)**	**0.01**
				Mixed site	-0.23	0.48	0.80 (0.31-2.05)	0.64
			RCVS Specialist	No RCVS specialist on site	-	-	1.00	-
				**RCVS specialist on site**	**0.74**	**0.20**	**2.10 (1.42-3.08)**	**<0.01**
			Sampling type / site	Urine	-	-	1.00	-
				**Anal region (including anal sacs)**	**0.40**	**0.11**	**1.49 (1.21-1.84)**	**<0.01**
				**Ear(s)**	**0.56**	**0.13**	**1.75 (1.36-2.25)**	**<0.01**
				Faeces	-0.58	0.43	0.56 (0.24-1.30)	0.18
				**Oronasopharyngeal & respiratory**	**0.53**	**0.21**	**1.71 (1.12-2.59)**	**0.01**
				**Other sites or mixed**	**0.55**	**0.09**	**1.74 (1.46-2.07)**	**<0.01**
				**Cats**				
Practice	26.20	(5.12)	Intercept		-7.68	0.60	0.00 (0.00-0.00)	
			Genetic breed group [g]	West Europe	-	-	1.00	-
				Asian	-0.85	0.55	0.43 (0.15-1.26)	0.12
				Crossbreed	-0.71	0.37	0.49 (0.24-1.01)	0.05
				**Not yet genetically classified**	**-2.01**	**0.83**	**0.13 (0.03-0.68)**	**0.02**
				Unknown breed	-0.61	0.49	0.54 (0.21-1.41)	0.21
			RCVS Specialist	No RCVS specialist on site	-	-	1.00	-
				RCVS specialist on site	1.24	0.75	3.46 (0.79-15.18)	0.10
			Sampling type / site	Urine	-	-	1.00	-
				**Anal region (including anal sacs)**	**1.45**	**0.67**	**4.27 (1.16-15.72)**	**0.03**
				Ear(s)	1.12	0.70	3.05 (0.77-12.10)	0.11
				Faeces	0.51	0.89	1.66 (0.29-9.53)	0.57
				Oronasopharyngeal & respiratory	0.37	0.59	1.45 (0.45-4.64)	0.53
				**Other sites or mixed**	**0.81**	**0.33**	**2.24 (1.18-4.26)**	**0.01**

[a] Variance
[b] Standard deviation
[c] Standard error
[d] Odds ratio
[e] 95% Confidence interval
[f] Vonholdt et al. (2010)
[g] Lipinski et al. (2008)

Supplementary material 13: Potentiated penicillin resistance. Multivariable mixed effects logistic models displaying risk factors significantly associated with odds of an *E. coli* clinical isolate being classed as potentiated penicillin resistant in dogs and cats. For both models, veterinary practice site and laboratory site were modelled as random effects. Significant categories within a variable are emboldened.

Random effect(s)		Fixed effects					
**Variable**	**Var [a] (SD) [b]**	**Variable**	**Category**	**β**	**SE [c]**	**OR [d] (CI) [e]**	***P***
			**Dogs**				
Practice	0.10 (0.31)	Intercept		-1.93	0.18	0.15 (0.10-0.21)	
Laboratory	0.09 (0.29)	Sex	Female	-	-	1.00	-
			Male	-0.08	0.05	0.93 (0.84-1.02)	0.11
			Unspecified	0.19	0.13	1.20 (0.94-1.54)	0.14
		Genetic breed Group [f]	Retriever	-	-	1.00	-
			Ancient / spitz	0.16	0.18	1.18 (0.84-1.66)	0.35
			Crossbreed	0.10	0.09	1.10 (0.93-1.30)	0.27
			Herding	-0.15	0.13	0.87 (0.68-1.11)	0.25
			**Mastiff-like**	**0.32**	**0.09**	**1.38 (1.16-1.65)**	**<0.01**
			Scent hound	0.26	0.14	1.30 (1.00-1.70)	0.05
			Sight hound	0.00	0.19	1.00 (0.69-1.46)	1.00
			**Small terrier**	**0.30**	**0.10**	**1.35 (1.11-1.65)**	**<0.01**
			Spaniel	0.13	0.09	1.13 (0.96-1.34)	0.14
			**Toy**	**0.38**	**0.13**	**1.47 (1.14-1.89)**	**<0.01**
			Not yet genetically classified	0.17	0.09	1.18 (0.99-1.41)	0.07
			Unknown breed	0.12	0.10	1.13 (0.93-1.37)	0.22
			**Working dog**	**0.29**	**0.11**	**1.34 (1.08-1.67)**	**0.01**
		Species treated	Dog & cat	-	-	1.00	-
			Dog, cat & equine	0.09	0.15	1.10 (0.82-1.46)	0.54
			**Dog, cat, equine & farm**	**-0.16**	**0.08**	**0.86 (0.74-1.00)**	**0.04**
			Dog, cat & farm	0.15	0.14	1.16 (0.89-1.51)	0.26
		Referrals only	Not referrals-only site	-	-	1.00	-
			**Referrals-only site**	**0.54**	**0.17**	**1.71 (1.21-2.40)**	**<0.01**
			Mixed site	-0.37	0.27	0.69 (0.41-1.17)	0.17
		RCVS Specialist	No RCVS specialist on site	-	-	1.00	-
			**RCVS specialist on site**	**0.31**	**0.10**	**1.37 (1.12-1.67)**	**<0.01**
		Sampling type / site	Urine	-	-	1.00	-
			**Anal region (including anal sacs)**	**0.18**	**0.07**	**1.20 (1.04-1.38)**	**0.01**
			Ear(s)	-0.02	0.10	0.98 (0.81-1.18)	0.80
			Faeces	0.07	0.22	1.07 (0.70-1.64)	0.77
			**Oronasopharyngeal & respiratory**	**0.43**	**0.14**	**1.53 (1.16-2.03)**	**<0.01**
			**Other sites or mixed**	**0.64**	**0.06**	**1.90 (1.70-2.12)**	**<0.01**
			**Cats**				
Practice	0.37 (0.61)	Intercept		-1.88	0.44	0.15 (0.07-0.36)	
Laboratory	0.65 (0.81)	Sex	Female	-	-	1.00	-
			Male	0.15	0.08	1.16 (1.00-1.36)	0.06
			Unspecified	0.14	0.20	1.15 (0.77-1.72)	0.49
		Sites per postcode	Single site	-	-	1.00	-
			Multiple sites	0.40	0.21	1.49 (0.98-2.25)	0.06
		Sampling type / site	Urine	-	-	1.00	-
			Anal region (including anal sacs)	-0.13	0.32	0.88 (0.47-1.65)	0.68
			Ear(s)	-0.41	0.34	0.66 (0.34-1.28)	0.22
			Faeces	-0.21	0.32	0.81 (0.44-1.51)	0.51
			Oronasopharyngeal & respiratory	0.37	0.20	1.44 (0.97-2.14)	0.07
			**Other sites or mixed**	**0.50**	**0.12**	**1.65 (1.30-2.09)**	**<0.01**

[a] Variance
[b] Standard deviation
[c] Standard error
[d] Odds ratio
[e] 95% Confidence interval
[f] Vonholdt et al. (2010)
[g] Lipinski et al. (2008)

Supplementary material 14: Coverage map displaying the percentage of veterinary practice sites contributing at least one *Enterobacteriaceae* AST result to this study by postcode area (*n*=127), displayed as a percentage of total practice sites contained within a postcode area, as recorded on the RCVS practice register. Histogram displays a count of postcode area sites displayed against practice site percentage coverage.

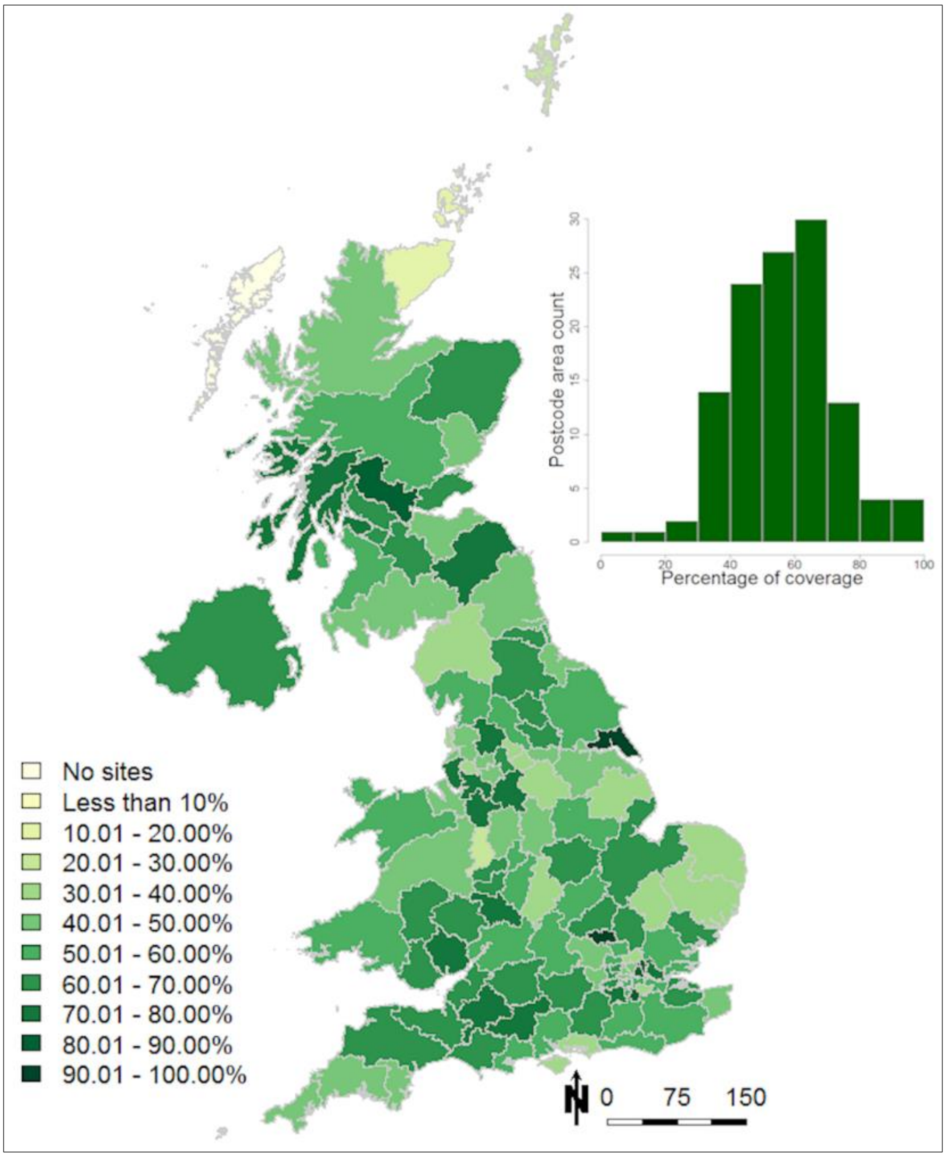

Supplementary material 15: Demographic features pertaining to *Enterobacteriaceae* clinical antimicrobial susceptibility test data provided by four veterinary diagnostic laboratories, originating from dogs (n=29,330) and cats (n=8,279).

	Dog		Cat	
**Variable**	**Category**	**% [a] (CI) [b]**	**Category**	**% [a] (CI) [b]**
Sex	Female	58.8 (57.9-59.7)	Female	59.5 (57.9-61.0)
	Male	37.9 (37.0-38.7)	Male	36.4 (35.0-37.7)
	Unspecified	3.3 (2.7-4.0)	Unspecified	4.2 (3.0-5.4)
Neutered	Neutered	60.2 (58.9-61.4)	Neutered	77.7 (75.8-79.8)
	Entire	20.9 (19.9-21.9)	Entire	8.4 (7.5-9.4)
	Unspecified	18.9 (17.4-20.4)	Unspecified	13.8 (11.8-15.8)
Genetic breed group [c]	Ancient / spitz	2.2 (2.0-2.4)	Asian	6.0 (5.4-6.6)
	Crossbreed	13.7 (13.2-14.3)	Crossbreed	71.1 (69.5-72.7)
	Herding	4.6 (4.3-4.9)	Mediterranean	0.1 (0.04-0.2)
	Mastiff-like	10.7 (10.3-11.2)	Unclassified	3.9 (3.4-4.4)
	Retriever	14.3 (13.8-14.9)	Unknown	12.7 (11.4-14.0)
	Scent hound	3.1 (2.9-3.4)	West Europe	6.2 (5.6-6.8)
	Sight hound	1.6 (1.5-1.8)		
	Small terrier	6.8 (6.5-7.2)		
	Spaniel	14.1 (13.6-14.6)		
	Toy	4.4 (4.1-4.7)		
	Unclassified	9.9 (9.4-10.3)		
	Unknown	9.5 (8.7-10.3)		
	Working dog	5.0 (4.7-5.3)		
Sample site/type	Urine	55.2 (54.0-56.3)	Urine	80.3 (78.9-81.7)
	Anal region [d]	11.0 (10.3-11.8)	Anal region [d]	1.4 (1.2-1.7)
	Ear(s)	12.1 (11.5-12.7)	Ear(s)	2.6 (2.2-3.0)
	Faeces	2.9 (3.7-3.2)	Faeces	2.3 (1.9-2.6)
	OP & respiratory [e]	2.1 (1.9-2.3)	OP & respiratory [e]	3.3 (2.9-3.8)
	Other/mixed sites	16.7 (15.9-17.5	Other/mixed sites	10.1 (9.1-11.1).

[a] Percentage of antimicrobial susceptibility test results

[b] 95% Confidence interval

[c] As defined for dogs (Vonholdt et al., 2010) and cats (Lipinski et al., 2008).

[d] Including anal sacs

[e] oropharyngeal

Supplementary material 16: Summary of clinical *Enterobacteriaceae* species isolated with completed antimicrobial susceptibility tests for dogs (*n*=29,330) and cats (*n*=8,279).

Bacterial species	Dogs		Cats	
	Number of AST results	%	Number of AST results	%
Escherichia coli	20343	69.359	7497	90.554
Proteus mirabilis	5326	18.159	268	3.237
Salmonella species	646	2.203	64	0.773
Enterobacter cloacae	623	2.124	161	1.945
Proteus species	619	2.110	25	0.302
Klebsiella pneumoniae	483	1.647	48	0.580
Pantoea agglomerans	239	0.815	28	0.338
Serratia marcescens	169	0.576	40	0.483
Enterobacter gergoviae	158	0.539	2	0.024
Klebsiella oxytoca	137	0.467	38	0.459
Citrobacter koseri	108	0.368	7	0.085
Klebsiella species	62	0.211	5	0.060
Enterobacter asburiae	36	0.123	10	0.121
Serratia liquefaciens	36	0.123	15	0.181
Morganella morganii	35	0.119	9	0.109
Enterobacter aerogenes	31	0.106	6	0.072
Proteus vulgaris	30	0.102	4	0.048
Citrobacter freundii	28	0.095	5	0.060
Leclercia adecarboxylata	24	0.082	2	0.024
Citrobacter braakii	18	0.061	6	0.072
Proteus hauseri	16	0.055	5	0.060
Providencia rettgeri	15	0.051	5	0.060
Raoultella ornithinolytica	14	0.048	3	0.036
Serratia fonticola	13	0.044	2	0.024
Escherichia vulneris	10	0.034		
Hafnia alvei	9	0.031	3	0.036
Providencia stuartii	9	0.031		
Enterobacter amnigenus	8	0.027		
Pantoea species	7	0.024	1	0.012
Enterobacter ludwigii	6	0.020	1	0.012
Enterobacter species	6	0.020	2	0.024
Enterobacter kobei	5	0.017		
Klebsiella variicola	5	0.017		
Proteus penneri	5	0.017	1	0.012
Raoultella planticola	5	0.017	1	0.012
Serratia grimesii	4	0.014	2	0.024
Serratia rubidaea	4	0.014	1	0.012
Escherichia albertii	3	0.010	1	0.012
Kluyvera intermedia	3	0.010		
Serratia species	3	0.010	2	0.024
Citrobacter amalonaticus	2	0.007		
Citrobacter gillenii	2	0.007		
Erwinia billingiae	2	0.007		
Pantoea septica	2	0.007	1	0.012
Rahnella aquatilis	2	0.007		
Serratia proteamaculans	2	0.007		
Serratia ureilytica	2	0.007	1	0.012
Buttiauxella gaviniae	1	0.003		
Citrobacter farmeri	1	0.003		
Citrobacter species	1	0.003		
Erwinia persicina	1	0.003		
Erwinia species	1	0.003		
Escherichia fergusonii	1	0.003		
Escherichia hermanii	1	0.003		
Ewingella americana	1	0.003	1	0.012
Lelliottia amnigena	1	0.003		
Moellerella species	1	0.003		
Pantoea dispersa	1	0.003	1	0.012
Providencia alcalifaciens	1	0.003	1	0.012
Raoultella species	1	0.003		
Raoultella terrigena	1	0.003		
Salmonella enterica	1	0.003	1	0.012
Citrobacter youngae			1	0.012
Rahnella species			1	0.012
Serratia odorifera			1	0.012

Supplementary material 17: Summary of clinical *Enterobacteriaceae* bacterial species groups isolated from dog and cat samples, by sample type / site.

Bacterial species groups	Sample type / site, % [a] (CI) [b]: Anal region (inc. anal sacs)	Ear(s)	Faeces	Oronasopharyngeal & respiratory	Other / mixed	Urine
**Dogs**						
Enterobacter spp.	1.1 (1.1-1.2)	27.8 (26.0-29.7)		5.0 (4.7-5.4)	47.2 (44.1-50.3	18.8 (17.5-20.0
Escherichia coli	13.3 (13.1-13.5)	7.3 (7.2-7.4)	1.3 (1.2-1.3)	2.0 (2.0-2.1)	17.5 (17.2-17.7)	58.6 (57.8-59.4)
Klebsiella pneumoniae	8.1 (7.4-8.8)	6.6 (6.0-7.2)	0.2 (0.2-0.2)	16.1 (14.7-17.6)	24.8 (22.6-27.1)	44.1 (40.2-48.0)
Other spp.	2.3 (7.7-8.1)	14.8 (13.9-15.7)		5.0 (4.7-5.3)	43.8 (41.1-46.6)	34.1 (32.0-36.2)
Proteus mirabilis	7.9 (7.7-8.1)	24.9 (24.2-25.5)		0.2 (0.2-0.2)	5.1 (5.0-5.3)	62.0 (60.3-63.6)
Other *Proteus* spp.	6.0 (5.5-6.4)	48.7 (45.0-52.3)		0.9 (0.8-1.0	10.7 (9.9-11.6)	33.7 (31.2-36.3)
Salmonella spp.			92.9 (85.7-100.0)	0.3 (0.3-0.3)	4.6 (4.3-5.0)	2.2 (2.0-2.3)
**Cats**						
Enterobacter spp.		6.0 (0.5-0.7)		12.6 (10.9-14.5)	49.5 (42.2-56.6)	31.9 (27.2-36.5)
Escherichia coli	1.5 (1.4-1.5)	1.8 (1.7-1.8)	1.7 (1.7-1.8)	2.9 (2.8-3.0)	8.3 (8.1-8.5)	83.8 (81.9-85.7)
Klebsiella pneumoniae	2.1 (1.5-2.7)	10.4 (7.5-13.4)	2.1 (1.5-2.7)	12.5 (9.0-16.0)	37.5 (26.9-48.1)	35.4 (25.4-45.4)
Other spp.	1.1 (0.9-1.2)	13.6 (11.6-15.6)		12.0 (10.2-13.7)	36.4 (31.2-41.7)	37.0 (31.6-42.3)
Proteus mirabilis	1.9 (1.6-2.1)	13.8 (12.2-15.5)		0.7 (0.7-0.8)	9.7 (8.5-10.9)	73.9 (65.0-82.7)
Other *Proteus* spp.	2.9 (1.9-3.8)	8.6 (5.7-11.4)		2.9 (1.9-3.8)	25.7 (17.2-34.2)	60.0 (40.1-79.9)
Salmonella spp.			87.7 (66.4-100.0)	3.1 (2.3-3.8)	6.2 (4.7-7.6)	3.1 (2.3-3.8)

[a] Percentage of antimicrobial susceptibility test results, by bacterial species groups

[b] 95% Confidence interval

Supplementary material 18: Univariable results from a mixed effects logistic regression model, exploring odds of 3rd/4th generation cephalosporin resistance in *E. coli* at an isolate-level against a range of putative risk factors for dogs. Veterinary practice site and diagnostic laboratory site was included as a random effect in all models.

**Variable**	**Category**	**β**	**SE** [a]	**OR** [b] **(CI)** [c]	***P***
Sex	Female (Intercept)	-1.76	0.71	0.17 (0.04-0.69)	
	Male	-0.06	0.06	0.94 (0.84-1.06)	0.31
	Unspecified	0.28	0.16	1.33 (0.98-1.80)	0.07
Neuter status	Un-neutered (Intercept)	-1.80	0.70	0.17 (0.04-0.66)	
	Neutered	-0.05	0.07	0.95 (0.83-1.10)	0.50
	Unspecified	0.18	0.10	1.19 (0.99-1.43)	0.07
Genetic breed group [d]	Retriever (Intercept)	-2.00	0.73	0.14 (0.03-0.57)	
	Ancient / spitz	0.50	0.22	1.57 (1.02-2.40)	0.04
	Crossbreed	0.28	0.11	1.33 (1.07-1.65)	0.01
	Herding	-0.18	0.16	0.84 (0.61-1.15)	0.27
	Mastiff-like	0.37	0.12	1.45 (1.15-1.84)	<0.01
	Scent hound	0.49	0.17	1.63 (1.18-2.27)	<0.01
	Sight hound	-0.34	0.28	0.72 (0.41-1.25)	0.24
	Small terrier	0.43	0.13	1.53 (1.18-1.98)	<0.01
	Spaniel	0.26	0.11	1.29 (1.04-1.60)	0.02
	Toy	0.47	0.17	1.60 (1.15-2.21)	<0.01
	Not yet genetically classified	0.14	0.12	1.15 (0.90-1.46)	0.27
	Unknown breed	0.25	0.12	1.28 (1.01-1.63)	0.05
	Working dog	0.50	0.14	1.65 (1.24-2.19)	<0.01
Urban / rural status	Urban (Intercept)	-1.75	0.71	0.17 (0.04-0.71)	
	Rural	-0.11	0.09	0.90 (0.76-1.07)	0.23
Species treated	Dog & cat (Intercept)	-1.77	0.70	0.17 (0.04-0.68)	0.01
	Dog, cat & equine	0.21	0.20	1.24 (0.84-1.83)	0.29
	Dog, cat, equine & farm	-0.22	0.11	0.80 (0.65-0.99)	0.04
	Dog, cat & farm	0.09	0.20	1.09 (0.74-1.61)	0.66
RCVS accreditation	Not accredited (Intercept)	-1.87	0.71	0.16 (0.04-0.62)	
	Accredited	0.16	0.07	1.17 (1.03-1.34)	0.02
RCVS hospital status	Not hospital (Intercept)	-1.83	0.70	0.16 (0.04-0.63)	
	Hospital	0.18	0.10	1.19 (0.99-1.44)	0.07
Emergency / out of hours provider	Not emergency / OOH (Intercept)	-1.79	0.71	0.17 (0.04-0.67)	
	Emergency / OOH provider	0.11	0.16	1.12 (0.82-1.52)	0.47
Referrals only	Not referrals-only site (Intercept)	-1.86	0.64	0.16 (0.04-0.55)	
	Referrals-only site	1.17	0.26	3.23 (1.96-5.34)	<0.01
	Mixed site	-0.24	0.33	0.79 (0.41-1.51)	0.47
RCVS AVP [e]	No RCVS AVPs on site (Intercept)	-1.81	0.70	0.16 (0.04-0.64)	
	RCVS AVPs on site	0.09	0.08	1.10 (0.94-1.29)	0.25
RCVS Specialist	No RCVS specialist on site (Intercept)	-1.94	0.61	0.14 (0.04-0.48)	
	RCVS specialist on site	0.66	0.14	1.93 (1.47-2.54)	<0.01
Veterinary nurse training	Not VN training site (Intercept)	-1.85	0.71	0.16 (0.04-0.63)	
	VN training site	0.09	0.09	1.09 (0.91-1.30)	0.34
Sites per postcode	Single site (Intercept)	-1.78	0.71	0.17 (0.04-0.68)	
	Multiple sites	-0.09	0.19	0.92 (0.64-1.32)	0.64
Sampling type / site	Urine (Intercept)	-2.05	0.68	0.13 (0.03-0.49)	
	Anal region (including anal sacs)	0.27	0.08	1.31 (1.11-1.54)	<0.01
	Ear(s)	0.77	0.57	2.16 (0.70-6.63)	0.18
	Faeces	0.08	0.25	1.08 (0.66-1.77)	0.76
	Oronasopharyngeal & respiratory	0.11	0.22	1.11 (0.72-1.70)	0.63
	Other sites or mixed	0.64	0.07	1.90 (1.61-2.18)	<0.01

[a] Standard error
[b] Odds ratio
[c] 95% Confidence interval
[d] Vonholdt et al. (2010)
[e] Royal College of Veterinary Surgeons Advanced Veterinary Practitioner

Supplementary information 19: Univariable results from a mixed effects logistic regression model, exploring odds of 3rd/4th generation cephalosporin resistance in *E. coli* at an isolate-level against a range of putative risk factors for cats. Veterinary practice site and diagnostic laboratory site was included as a random effect in all models.

Variable	Category	β	SE [a]	OR [b] (CI) [c]	*P*
Sex	Female (Intercept)	-3.01	0.10	0.05 (0.04-0.06)	
	Male	0.34	0.11	1.40 (1.14-1.73)	<0.01
	Unspecified	0.12	0.30	1.13 (0.63-2.03)	0.69
Neuter status	Un-neutered (Intercept)	-3.10	0.21	0.05 (0.03-0.07)	
	Neutered	0.27	0.20	1.31 (0.88-1.94)	0.18
	Unspecified	0.13	0.26	1.14 (0.69-1.89)	0.61
Genetic breed group [d]	West Europe (Intercept)	-2.80	0.21	0.06 (0.04-0.09)	
	Asian	-0.03	0.29	0.97 (0.55-1.72)	0.92
	Crossbreed	0.02	0.21	1.02 (0.68-1.54)	0.93
	Not yet genetically classified	-0.49	0.37	0.62 (0.30-1.27)	0.19
	Unknown breed	-0.47	0.26	0.63 (0.38-1.05)	0.08
Urban / rural status	Urban (Intercept)	-2.87	0.10	0.06 (0.05-0.07)	
	Rural	0.01	0.18	1.01 (0.71-1.43)	0.98
Species treated	Dog & cat (Intercept)	-2.87	0.09	0.06 (0.05-0.07)	
	Dog, cat & equine	0.02	0.39	1.02 (0.47-2.20)	0.96
	Dog, cat, equine & farm	0.15	0.21	1.16 (0.76-1.77)	0.48
	Dog, cat & farm	-0.47	0.45	0.62 (0.26-1.49)	0.29
RCVS accreditation	Not accredited (Intercept)	-2.99	0.12	0.05 (0.04-0.06)	
	Accredited	0.22	0.13	1.25 (0.97-1.59)	0.08
RCVS hospital status	Not hospital (Intercept)	-2.86	0.10	0.06 (0.05-0.07)	
	Hospital	-0.04	0.18	0.96 (0.67-1.37)	0.83
Emergency / out of hours provider	Not emergency / OOH (Intercept)	-2.87	0.09	0.06 (0.05-0.07)	
	Emergency / OOH provider	0.10	0.29	1.10 (0.62-1.94)	0.74
Referrals only	Not referrals-only site (Intercept)	-2.89	0.09	0.06 (0.05-0.07)	
	Referrals-only site	0.78	0.49	2.17 (0.82-5.72)	0.12
	Mixed site	0.12	0.55	1.13 (0.39-3.33)	0.82
RCVS AVP [e]	No RCVS AVPs on site (Intercept)	-2.87	0.10	0.06 (0.05-0.07)	
	RCVS AVPs on site	0.02	0.15	1.02 (0.76-1.38)	0.95
RCVS Specialist	No RCVS specialist on site (Intercept)	-2.89	0.09	0.06 (0.05-0.07)	
	RCVS specialist on site	0.48	0.26	1.61 (0.97-2.69)	0.07
Veterinary nurse training	Not VN training site (Intercept)	-2.76	0.16	0.06 (0.05-0.09)	
	VN training site	-0.13	0.16	0.88 (0.65-1.21)	0.44
Sites per postcode	Single site (Intercept)	-2.88	0.09	0.06 (0.05-0.07)	
	Multiple sites	0.35	0.30	1.42 (0.79-2.55)	0.24
Sampling type / site	Urine (Intercept)	-2.93	0.09	0.05 (0.05-0.06)	
	Anal region (including anal sacs)	0.28	0.37	1.32 (0.63-2.75)	0.46
	Faeces	-1.06	0.60	0.35 (0.11-1.12)	0.08
	Oronasopharyngeal & respiratory	0.73	0.25	2.07 (1.28-3.37)	<0.01
	Other sites or mixed	0.65	0.16	1.91 (1.39-2.63)	<0.01

[a] Standard error
[b] Odds ratio
[c] 95% Confidence interval
[d] Lipinski et al. (2008)
[e] Royal College of Veterinary Surgeons Advanced Veterinary Practitioner
